# Molecular evolution of the ependymin protein family: a necessary update

**DOI:** 10.1186/1471-2148-7-23

**Published:** 2007-02-15

**Authors:** Edna C Suárez-Castillo, José E García-Arrarás

**Affiliations:** 1 Department of Biology, University of Puerto Rico, Río Piedras Campus, 00931, Puerto Rico

## Abstract

**Background:**

Ependymin (Epd), the predominant protein in the cerebrospinal fluid of teleost fishes, was originally associated with neuroplasticity and regeneration. Ependymin-related proteins (Epdrs) have been identified in other vertebrates, including amphibians and mammals. Recently, we reported the identification and characterization of an Epdr in echinoderms, showing that there are ependymin family members in non-vertebrate deuterostomes. We have now explored multiple databases to find Epdrs in different metazoan species. Using these sequences we have performed genome mapping, molecular phylogenetic analyses using Maximum Likelihood and Bayesian methods, and statistical tests of tree topologies, to ascertain the phylogenetic relationship among ependymin proteins.

**Results:**

Our results demonstrate that ependymin genes are also present in protostomes. In addition, as a result of the putative fish-specific genome duplication event and posterior divergence, the ependymin family can be divided into four groups according to their amino acid composition and branching pattern in the gene tree: 1) a brain-specific group of ependymin sequences that is unique to teleost fishes and encompasses the originally described ependymin; 2) a group expressed in non-brain tissue in fishes; 3) a group expressed in several tissues that appears to be deuterostome-specific, and 4) a group found in invertebrate deuterostomes and protostomes, with a broad pattern of expression and that probably represents the evolutionary origin of the ependymins. Using codon-substitution models to statistically assess the selective pressures acting over the ependymin protein family, we found evidence of episodic positive Darwinian selection and relaxed selective constraints in each one of the postduplication branches of the gene tree. However, purifying selection (with among-site variability) appears to be the main influence on the evolution of each subgroup within the family. Functional divergence among the ependymin paralog groups is well supported and several amino acid positions are predicted to be critical for this divergence.

**Conclusion:**

Ependymin proteins are present in vertebrates, invertebrate deuterostomes, and protostomes. Overall, our analyses suggest that the ependymin protein family is a suitable target to experimentally test subfunctionalization in gene copies that originated after gene or genome duplication events.

## Background

Ependymin (Epd), a glycoprotein that is found in high concentrations within the cerebrospinal fluid of teleost fishes was originally associated with learning processes in fishes [1]. Although the original protein sequence was obtained in the 1980's by classical methods of protein purification, with the advent of molecular biology the *epd *genes of many fish species were sequenced [2-6]. *epd *sequences have been determined to be a useful tool for studying the phylogeny of teleost fishes [6], based on high conservation of piscine sequences in certain regions (i.e., in the overall protein domain, specially near the potential glycosylation sites) and high divergence at others (i.e., at the amino and carboxyl ends) while retaining enough phylogenetic signal to resolve with confidence the relationship among distantly related taxa.

More recently ependymin-related proteins (Epdrs) have been identified in other vertebrates including amphibians and mammals [7,8]. Since Epds and Epdrs had been found only in vertebrate species, this protein family was considered unique to this group [9-11]. However, the discovery by our group of an *epdr *gene in echinoderms showed that the family extends to non-vertebrate deuterostomes and questioned whether Epds could be a deuterostome specific protein family [12].

Differences in sequence and tissue expression between fishes and mammals also surfaced in our previous work. Whereas in fishes the known Epd proteins are brain-specific [13-15], in mammals the Epd protein is expressed in several different tissues [7,8]. Moreover, according to our initial phylogenetic analyses, the echinoderm sequences clustered with those from mammals and the *epd *transcript showed a broad pattern of expression in echinoderm tissues [12].

The availability of new sequence data from piscine expressed sequence tags (ESTs) projects, allowed us to identify additional *epd *members that interestingly were isolated from non-brain tissue. Some of these "new" *epd *sequences came from fishes such as zebrafish, whose brain-specific *epd *gene had been well characterized [16]. Zebrafish non-brain derived *epd *ESTs showed significant differences (at the expression and amino acid levels) from the previously known brain associated *epd*. This fact suggested that the Epd protein family was larger and more complex than previously thought, and encouraged us to undertake a systematic search in multiple databases for new members of this protein family in different metazoans. Our focus was twofold. First, we investigated if the non-brain specific piscine Epd proteins were evolutionarily more related to the previously known brain-specific piscine sequences, or if, as suggested by their broader expression pattern, they were closer to mammal and echinoderm Epds. Second, we looked for the presence of Epd domain-containing proteins in protostomes.

New Epd proteins were identified in our survey from multiple metazoan ESTs and genome databases. Using these sequences and all the previously well-known *epds*, we assembled the most comprehensive Epd dataset to date for phylogenetic analyses and assessment of natural selection pressure. It was in this context that we attempted to determine what relationship, if any, exists between the evolutionary history of this gene family and tissue-specific patterns of gene expression.

Our findings clearly demonstrate that Epd domain-containing proteins are present in vertebrates, invertebrate deuterostomes, and protostomes. In addition, our phylogenetic analyses suggest that two fish-specific genome duplications shaped the evolution of the Epds. The resulting gene duplicates have undergone functional divergence either by positive selection or relaxed purifying selection, as indicated by an increased rate of nonsynonymous substitution. Based on our evolutionary analyses we classified Epds into four protein groups. The absence in tetrapods of two of these groups provides further support for fish-specific genome duplications [17-20] that resulted in the appearance of teleost-specific duplicate genes. Our data suggest that a combination of the duplication-degeneration-complementation (DDC) model [21,22] and adaptive evolution in the coding regions [23] contributed to evolution of both teleost-specific gene copies and a deuterostome-specific Epd protein group. Our analyses identify several codon/amino acid sites that appear to account for the escape from pseudogenization of these genes and their gaining of rapid functional divergence [24,25].

The results presented here provide some important clues to the functional differences of Epds. While the originally described brain-specific Epds have been associated with neuroplasticity and optic nerve regeneration in fishes [1], the mammalian Epds are expressed in a variety of tissues and specifically the human e*pd *have been shown to be overexpressed in colon cancer [8] and in hematopoietic stem/progenitor cells just before the onset of proliferation and differentiation [7]. Moreover, our own studies [12] demonstrated that the echinoderm *epds *are expressed in several tissues and its overexpression suggested its involvement in intestinal regeneration. Thus, it seems likely that the structural differences shown here to occur among Epds, that serve to group them in evolutionary terms, can also be used to identify their particular functions in the tissues, organs and species where they are expressed.

## Results and Discussion

### Identification of new members of the ependymin protein family

The InterPro [26] Epd protein family (IPR001299) is composed of protein sequences bearing only the Epd domain. The architecture and signatures defining this domain are very distinctive [6,7,12]. The fact that there are no domains/families known to be related to [InterPro: IPR001299] means that a protein can be assigned to the Epd family if it displays the sequence patterns established for the domain.

Taking advantage of recently sequenced genomes and ongoing EST projects, we were able to find 39 new members of the Epd protein family [see additional file 1: Table_S1]. The assignment of each sequence to this family was corroborated by searches in the Conserved Protein Domains (CDD) database [27], detection of potential N-glycosylation sites [28], comparison of hydropathic profiles [29], prediction of cysteines participating in disulfide bond formation [30], and the presence of certain amino acids in conserved positions as compared to previously well-known Epd proteins. Most of the sequences passed every one of these tests. Cases where candidate ESTs possessed an N-terminal sequence were also verified by signal peptide screening [31]. All the newly identified *epd *sequences had significant similarity only with other Epd proteins as determined by Position-Specific Iterated Blast (PSI-BLAST) and standard BlastP searches [see additional file 2: Additional_Text for detailed methods].

Interestingly, four of the new *epd *sequences found during this study were from protostomes [see additional file 1: Table_S1]: the mollusks *Crassostrea gigas *(Oyster_Cgi), *Crassostrea virginica *(Oyster_Cvi),*Aplysia californica *(Aplysia), and *Biomphalaria glabrata *(Biomphala). The working names used here are given in parenthesis. The finding of Epd proteins in mollusks is remarkable since the Epd proteins were once thought to be chordate specific [9-11] and later deuterostome-specific [12]. The presence of Epd in mollusks clearly indicates that this family is older than previously thought and its origin can be traced at least to the origin of the protostomes. We failed to find Epd domain-containing proteins in other protostomes for which genome and EST data is available, such us *Drosophila *and *Caenorhabditis elegans*, but this does not rule out the possibility of finding additional family members in other protostomes once more metazoan species are sequenced. In addition, it has recently been proposed that model species such as *Drosophila *and *C. elegans *have suffered extensive gene loss during their natural history and that this hinders their utility in big scale genome comparisons [32]. An alternative explanation is that the Epd protein family is restricted to the deuterostomes and lophotrochozoans while absent from the ecdysozoans.

Additional new members of the Epd protein family were found in the taxonomic groups: Echinodermata, Urochordata, Cephalochordata, Amphibia, Aves, Elasmobranchii, and fishes [see additional file 1: Table_S1]. In the latter group, it is notable that this gene was previously known to be only expressed in the brain of teleost fishes [13-15]. However, we found several new members of the protein family expressed in other fish tissues that appear to be duplications of the previously described brain genes. Thus, besides the genes known to be expressed in brain, we found three additional *epd *genes in zebrafish and *Fugu*; two additional copies in *Tetraodon*, medaka, and salmon; and an additional copy in the catfish *Ictalurus punctatus*. All these new genes have a broader expression pattern (non-restricted to brain tissue), as can be inferred from the tissue source information deposited by the authors in the corresponding ESTs databases.

The 39 newly identified Epd proteins [see additional file 1: Table_S1] were combined with the 35 previously recognized members [see additional file 3: Table_S2] to produce an alignment of 74 members of the family which served as the basis for our molecular evolutionary analyses.

**Figure 1:**
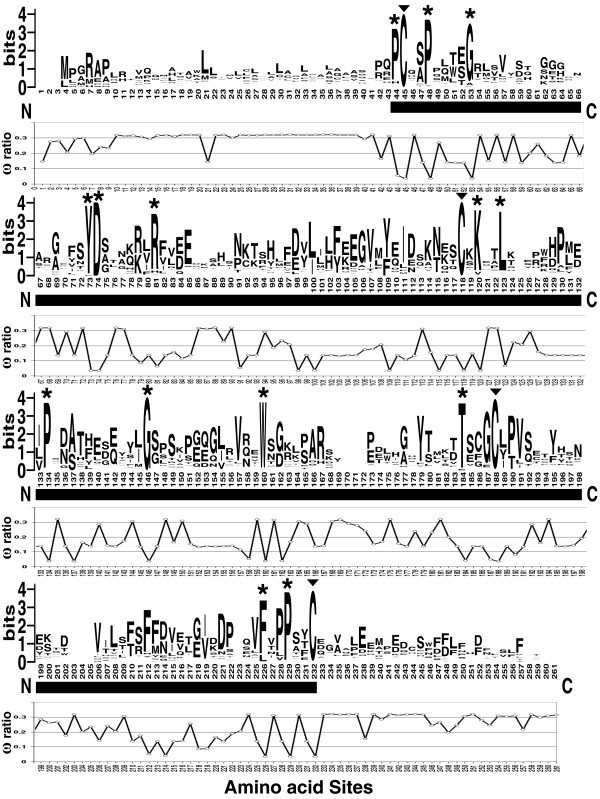
**Sequence logo representation of the ependymin protein family**. Overall comparison among all the 74 ependymin protein sequences used in this study in which the height of a given letter (amino acid residue) represents its frequency of occurrence at that particular site. High variability is observed in the amino (N) and carboxyl (C) terminals. Similarity among ependymins increases noticeably at the domain region (underlined with a solid black bar). Diagnostic cysteines putatively involved in disulfide bonding are indicated by arrowheads above the letter (▼). Asterisks (*) above the letters denote other invariable or almost invariable residues. Empty sites represent regions of the alignment where gaps were introduced in most of the sequences. The approximate posterior mean of the ω ratio (selective pressure) at each codon site calculated under model M3 (discrete) for all the ependymin proteins is shown below the WebLogo. The deeper furrows in the ω plot identify the amino acid sites that are under stronger purifying selection.

The protein sequence logo [33,34] representing the alignment of all the 74 Epd sequences (Figure 1) confirmed that amino (N) and carboxyl (C) ends are not well conserved, increasing similarity in the Epd domain region as pointed out in previous studies [6-8,12]. Inside the domain, several amino acids provided a hallmark of Epd molecules. The most relevant were four cysteines within the molecule primary sequence at positions 45, 118, 188 and 232 (numbered according to the WebLogo; Figure 1). These residues have also been predicted by the Disulfind server [30,35] to participate in disulfide bond formation, pointing to a key role in protein folding and/or dimeric interactions and ultimately to the biological function of the Epds [2,4,36]. Two proteins (Ictalur_GS and Danio_Tj) were found to lack one of these Cys but this was probably due to sequencing errors on these ESTs. Pro residues were usually found one to three residues from the Cys, particularly next to the first and fourth Cys. In addition, all Epds showed an Asp about 29 residues down from the initial Cys (D^74 ^in the WebLogo alignment; Figure 1). A Tyr residue (Y^73^) was found to precede the common Asp in all species except for echinoderms which showed a Phe at this position. Other residues common to Epds included: (i) the Gly (G^146^) at about 28 residues from the second Cys, (ii) the Pro (P^134^) at a halfway distance between the second Cys and the common Gly, and (iii) the Trp (W^160^), 11–14 residues after the common Gly. Only 3 species lack this Trp (Oysters have either a Tyr or a Phe, and the tunicate *Diplosoma listerianum *has a Tyr). Also notable was the high conservation of the residues P^44^, P^48^, G^53^, R^81^, K^120^, L^123^, T^184^, F^226^, and P^229^. In view of their high positional conservation, and the yet to be determined tridimensional structures of Epd proteins, we can only suggest that these amino acids are especially important to the overall Epd function.

**Figure 2:**
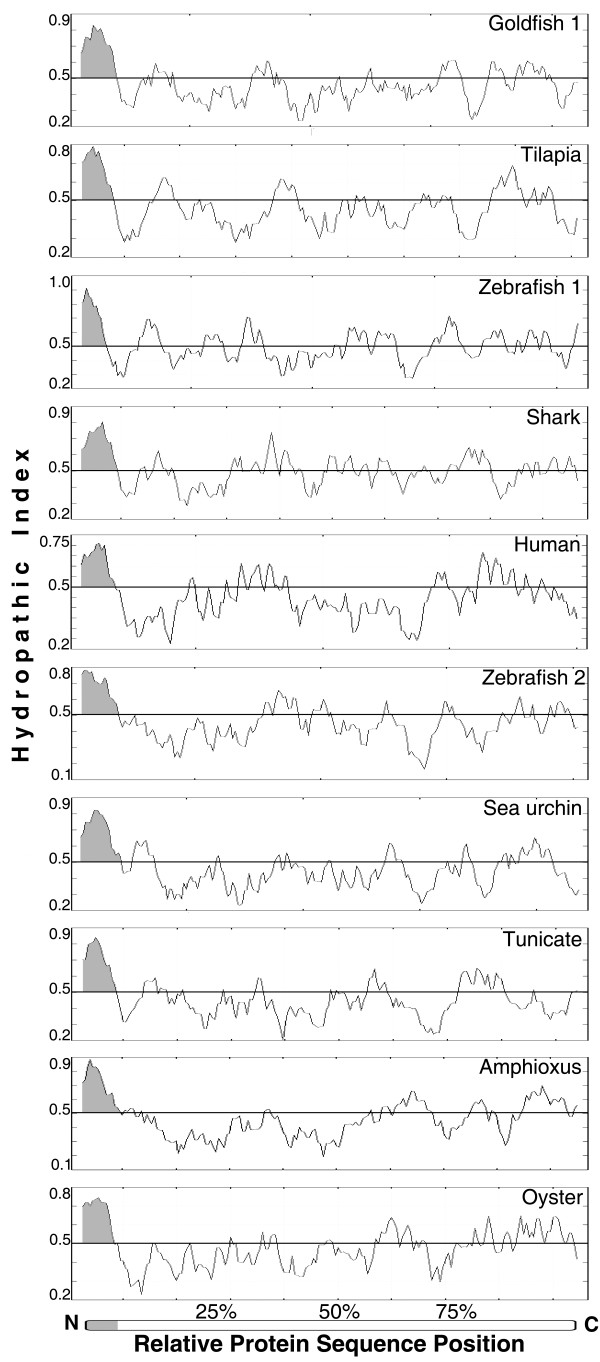
**Comparison of hydropathic profiles for selected ependymin proteins**. The hydropathic profiles of two previously recognized ependymin proteins, goldfish 1 (1Carassius) and human (Epdr1_Homo) are compared with the profiles of a subset of the new members of the ependymin protein family that we are reporting. The working names used in this study are given in parenthesis. Name equivalence is as follows: Zebrafish 1 (Dan_LvItEm), Zebrafish 2 (Danio_MERP), Shark (Shark_Squa), Tunicate (Diplo_Tun), Oyster (Oyster_Cvi). [See additional file 1: Table_S1 and additional file 3: Table_S2 for additional name identifiers such as GenBank accession numbers]. All sequences are complete except for the human in which the first 20 amino acids have been trimmed to facilitate the profile comparison with the other sequences. The vertical scale represents the hydropathic score for each amino acid and the horizontal scale shows the relative position in each protein. Scores above zero are considered hydrophobic while those below are considered hydrophilic. The relative position of the hydrophobic N-terminal signal peptide for each sequence is shaded.

The Epd protein family members can show highly divergent amino acid sequences but still maintain very similar hydropathy profiles, suggesting that the overall functional properties of the proteins are conserved [6]. These hydropathic profiles have been used to link Epd amino acid similarity with their properties as secreted glycoproteins [6-8,12]. As shown in Figure 2, the hydropathic profiles of Epd proteins are very similar. Thus, this family of proteins is mainly hydrophilic without transmembrane domains, but possesses a small, highly hydrophobic, region corresponding to the N-terminal signal peptide typical of secreted proteins.

### Virtual mapping

**Figure 3:**
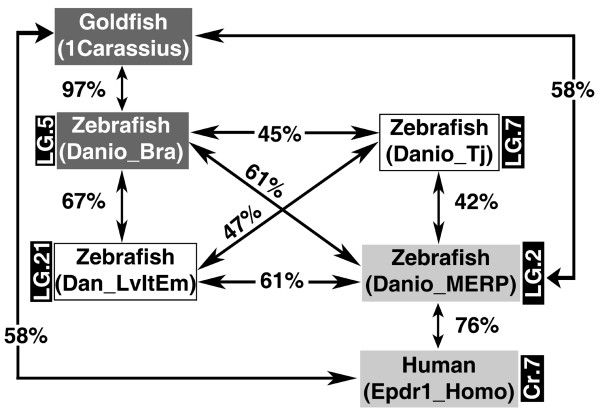
**Similarity relationship among the zebrafish ependymins and two previously known ependymin sequences**. The amino acid similarity of two zebrafish ependymins (Danio_Bra and Danio_MERP) is higher across species (e.g., goldfish and human) than among the four different zebrafish molecules. Dark gray boxes denote brain-specific sequences (Danio_Bra and 1Carassius), whereas white and light gray boxes indicate non-brain specific sequences (Danio_Tj, Dan_LvItEm, Danio_MERP, and Epdr1_Homo). The linkage group (LG) or chromosome (Cr) where the gene is present is indicated in the black box next to each sequence (this information is currently not available for goldfish).

Four *epd *sequences were found in the zebrafish genome, all of which mapped to different linkage groups (LG) in the zebrafish assembly Zv6. The first sequence was the previously well-known zebrafish *epd *gene *Danio_Bra *(mapped to the LG 5). The other zebrafish Epd genes analyzed were: *Danio_Tj *(LG 7), *Dan_LvItEm *(LG 21) and *Danio_MERP *(LG 2) [see additional file 4: Table_S3 for detailed results from mapping]. Two of the zebrafish protein sequences (Danio_Bra and Danio_MERP) had higher similarity to other vertebrate Epd sequences such as the goldfish and the human Epd protein than to the other zebrafish sequences (Figure 3). For instance, the amino acid similarity between the Danio_Bra and the goldfish Epd (1Carassius) expressed in brain was 97.2%, the similarity between the Danio_MERP and the human Epd protein (Epdr1_Homo) expressed in several tissues was 75.9%, while the similarity between Danio_Bra and Danio_MERP was only 60.5%.

Using genome and ESTs information from *Tetraodon*, we found that three different *epd *genes (*Tetraod_Br, Tetraod_Tj, and Tetrao_MEL*) mapped to different genomic locations [see additional file 4: Table_S3]. Pairwise comparisons of the three *Tetraodon *protein sequences yielded percentages of similarity ranging between 35% and 43%, much less than the similarity expected if they were the same gene. Since the *Tetraod_Br *and *Tetraod_Tj *genes were predicted from genome sequence and are not well supported by *Tetraodon *cDNAs (as is the case of the *Tetrao_MEL *gene), we have proposed that they are expressed by different tissues (i.e., brain and non-brain tissue), according to their location in the preliminary gene trees that we generated from the gathered data.

We found four *Fugu *Epd sequences (*Fugu_Brain, Fugu_HerGi, Fugu_Tj*, and *Fugu_MERP*) localized to different scaffolds of the current *Fugu *genome assembly [see additional file 4: Table_S3]. Amino acid similarity in pairwise comparisons among them ranged from 50.7% to 66.1%.

Three different medaka Epd sequences (*Medaka_LW1*, *Medaka_LW2*, and *Medak_MERP*) were found and mapped to different scaffolds in the draft assembly of the medaka (*Oryzias latipes*) HdrR genome [see additional file 4: Table_S3]. Medaka_LW1 and Medaka_LW2 Epd proteins were 80% similar and only 46.5% identical, and each one of them differed from Medak_MERP by 41.3% and 43.11%, respectively.

Two *Ciona epd *domain-containing genes (*Ciona_Tun1, Ciona_Tun2*) were predicted from the *Ciona intestinalis *genome assembly [see additional file 4: Table_S3]. Although the two Ciona Epds were located on the same chromosome (12q), they were mapped to different regions and each one was encoded by several non-overlapping ESTs. The identity between the *Ciona *Epds was only 33%, providing further support for being different genes.

We were able to map only one *epd *gene for the frog (*Xenopus tropicalis*), chicken, human, mouse, rat, and chimpanzee genomes [see additional file 4: Table_S3]. Although two different mouse Epd proteins were available on the databases, our analyses indicated that they may not be two different genes [see additional file 2: Additional_Text for details on this issue]. We doubt that the *Epdr1_Mus *[GenBank:AY027861] is a mouse gene, but we included this sequence as well as the reliable mouse sequence *Epdr2_Mus *[GenBank:AF353717] in subsequent analyses since they are both currently acknowledged in GenBank as separate mouse *epd *genes.

### Phylogenetic distribution and relationships of ependymins

**Figure 4:**
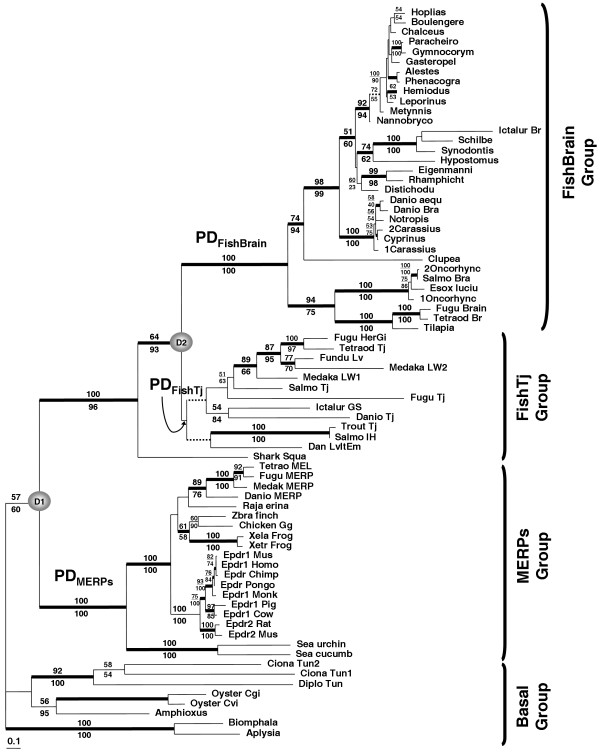
**Phylogenetic analysis of the ependymin protein family**. The results from a Maximum likelihood (ML) bootstrap analysis are shown above the branches, whereas the values below the branches result from a Neighbor-Joining (NJ) bootstrap analysis. The dashed and thick branches represent 91 – 92 % and >95% Bayesian posterior probability respectively. Only the ML and NJ bootstrap values >50% are shown. The labels on the tips correspond to the working names given to the sequences used in this study [see additional file 1: Table_S1 and additional file 3: Table_S2]. Gray circles indicate the putative duplication events studied (D_1 _and D_2_). For selective pressure analysis, the branches are partitioned into postduplication (PD; immediately postdating the gray circles) and postspeciation branches (PS; postdating divergence of the species). To facilitate interpretation of PD and PS branches, this tree has been rooted at the proposed Basal group composed by the mollusks, tunicates, and amphioxus. The other proposed groups are also indicated as PD and PS branches (i.e., PS_FishTj_, PS_FishBrain_, PS_MERPs_). All analyses were done using unrooted trees. The branch lengths are proportional to the number of substitutions per site (see scale bar in the figure).

Since previously published phylogenies included only 25 sequences expressed in fish brain [6], a few sequences from mammals, one from an amphibian [7], and three from echinoderms [12], our dataset of 74 family members represents a substantially more comprehensive sample of Epd diversity. Our results using various methods for phylogenetic inference (Bayesian, Maximum Likelihood, Neighbor-Joining, and Maximum Parsimony) showed essentially the same topology (Figure 4). The sole difference was that with maximum parsimony, several unresolved polytomies were obtained in the more derived branches within each main clade (data not shown). We note that bootstrap values and posterior probabilities were low for certain branches. However, an assumption for the bootstrapping method is even distribution of the phylogenetic signal throughout the data set [37]. In gene families like *epd*, with complete conservation of some sites and large divergence in others across the phylogeny, this assumption is not met. Thus, high bootstrap values are not always expected to be obtained for many nodes, because the sites supporting the existence of the main clades may differ from the sites that are useful for resolving the relationships among more derived groups [38].

Four clade groups could be inferred from our phylogenetic analyses (Figure 4): a first group that we have called "FishBrain" is composed of Epd proteins specifically expressed in teleost fish brain. The original Epd sequences were located in this group which has been the best studied group with more than 30 member sequences. In fact, most of the available Epd sequences from fishes came from an extensive study [6] which used brain tissue to clone orthologues of goldfish *epd *in other teleost fishes.

A second group that we named "FishTj" has remained unnoticed until now. This group is only present in fishes, but unlike the FishBrain group, its expression is not restricted to the brain. This group was represented by twelve Epd sequences that we placed as a putative sister clade to the FishBrain group (Figure 4). The FishTj group was composed of complete genes obtained from genome and ESTs sequencing projects in which the tissue was not brain derived. In fact, recently the EST sequences *Salmo_Tj *from *Salmo salar *and *Medaka_LW2 *from the medaka fish *Oryzias latipes *were shown to be upregulated in immunologically challenged liver: in the case of the salmonid sequence, after being exposed to the pathogen *Aeromonas salmonicida *[39], and in the case of medaka, after being exposed to the aryl hydrocarbon receptor agonist TCDD [40]. Thus, from the response displayed by these animals under toxic stress, we suggest that these FishTj Epds might be involved in repair following hepatic injury.

All the teleost fish species that have an *epd *gene in the FishTj group also have an unlinked *epd *copy in the FishBrain group (Figure 4). The only exception to this observation is the medaka where no brain-specific Epd protein was found, probably due to incomplete sequencing or assembling. In our results, the bootstrap value and posterior probability that support the FishTj group as a monophyletic clade are low, but the cohesion among the FishTj proteins is graphically strong. We predict that as more piscine *epd *sequences isolated from tissues other than brain become available, the support value for this group will raise dramatically.

It is interesting to note the position of the shark Epd sequence [Shark_Squa] (Figure 4). With the current information, this sequence was placed as the putative root of a big clade containing sequences only present in fishes that became divided into the FishBrain and FishTj paralog groups. Since this part of the tree resembles the duplication topology described by Meyer and colleagues [18], it could reflect the whole genome duplication postulated to have occurred during the natural history of teleost fishes [41]. Thus, it will be important to determine if the shark position remains unchanged after the addition of more sequences.

The monophyly of the third group that can be recognized from the tree (Figure 4) was highly supported. This group was composed of protein sequences isolated from echinoderms, amphibians, birds, mammals, an Elasmobranchii [Raja_erina], and four sequences from teleost fishes [Danio_MERP, Medak_MERP, Fugu_MERP, and Tetrao_MEL]. Since the first acknowledged member of this group was the human gene *epdr1 *formerly called *MERP1 *[7], we have named this clade the "MERPs" group. According to published experimental evidence [7,8,12], and to information deposited in the databases, these sequences were isolated from a variety of tissue sources, including but not restricted to brain, heart, skeletal muscle, prostate, kidney, liver, small intestine, colon, spleen and gonads in human and mouse, and in intestine, esophagus, mesenteries, gonads, respiratory trees, and tentacles in echinoderms. Clearly, the expression pattern of the Epd proteins in the MERPs group is not tissue-specific.

Although statistical support was poor (Figure 4), we also postulate the existence of a fourth Epd group that includes the evolutionary more basal species. This group, that we named "Basal", clusters *epd *genes from protostomes (mollusks) and deuterostomes (tunicates and amphioxus). Since all Basal group Epds are from invertebrates, we expected echinoderm sequences to fall within this group as well. Therefore, we performed the SH [42], KH and RELL [43] tests to compare the expected placement of echinoderm sequences within the Basal group as opposed to their placement inside the MERPs group observed in the inferred tree topology (Figure 4). However, all tests indicated significantly better support (*p *< 0.0001) for their assignment to the MERPs group. A notable misplacement in the Basal group was the location of the *epd *gene from oysters as sister to the amphioxus *epd *gene; instead of being sister group of the other mollusks: *Aplysia californica *[Aplysia] and *Biomphalaria glabrata *[Biomphala]. This misplacement (Figure 4) did not fit the data significantly better than the expected phylogenetic placement of the oysters as sisters of the other mollusks (*p*KH = 0.107, *p*SH = 0.112, *p*RELL = 0.101).

Interestingly, the *epd *sequence isolated from the oyster *Crassostrea gigas *[Oyster_Cgi] has been recently shown to be up-regulated in the digestive gland of these animals after a week of exposure to hydrocarbon contamination [44]. Since the digestive gland, similar to the liver, is known to plays major roles in metabolism and detoxification, this may imply that Epds in mollusks and vertebrates have conserved functions.

We expected that within each Epd paralog group, the species gene tree would be obtained after phylogenetic reconstruction. This was achieved for the FishBrain group where the branching pattern agreed considerably with previous studies focused on the phylogenetic relationship of teleost fishes [6,45,46]. In spite of the awkward, but statistically well supported positioning of the Epd sequences from echinoderms, the overall branching pattern inside the MERPs group also agreed satisfactorily with the expected species tree. However sampling bias against the metazoan groups that may carry genes belonging to any of the previously unidentified Epd groups (FishTj and Basal) appears to be the main impediment to obtain good statistical support for their monophyly and resolve the species relationships among the proteins inside each group. Thus, our reconstruction of the Epd phylogeny strongly agrees with the 2R hypothesis [41], providing a good example for the two rounds of genome duplications proposed to have occurred early in the vertebrate lineage; all exceptions found in the inferred topology are highlighted by low support values.

### Descriptive analysis of the ependymin protein family subgroups

To analyze the particular characteristics of each Epd group we selected only complete sequences with the initial methionine and the final stop codon. These included 13 Epds from the FishBrain group, 11 from the MERPs, 9 from the FishTj, and 6 from the Basal. Table 1 shows the major findings of these comparisons. If we use the Basal group to represent the putative original molecule it serves as a point of comparison on how the other group molecules have diverged. We are conscious that this Basal group contains species from three different phyla that might be quite distant from one another. Nonetheless, if we do the same analysis using only the two mollusk complete sequences, the variability they show between them is similar to that when compared with the other two phyla, thus essentially the same results are obtained.

**Table 1:** Quantitative survey of ependymin protein features (Average ± SE)

**Ependymin Group**	**Protein Length**^**a**^	**pI**^**b**^	**A**^**c**^	**B**^**d**^	**A/B**^**e**^	**AI**^**f**^	**Phe**^**g**^	**Trp**^**g**^	**Pro**^**g**^
FishBrain	217.1 ± 0.8	5.1 ± 0.1	29.3 ± 0.8	15.2 ± 0.7	2 ± 0.1	73.3 ± 2	13.8 ± 0.3	1.9 ± 0.1	10.2 ± 0.7
FishTj	214.3 ± 1.0	6.5 ± 0.4	20.8 ± 1.4	18.6 ± 1.1	1.2 ± 0.1	82.9 ± 2.7	13.8 ± 0.8	3.2 ± 0.3	13.7 ± 0.5
MERPs	222.2 ± 2.3	6.5 ± 0.3	23.7 ± 0.7	22.6 ± 0.4	1.1 ± 0.1	74.3 ± 1.2	7.7 ± 0.5	6.6 ± 0.4	15.4 ± 0.5
Basal	197.2 ± 2.6	5.5 ± 0.3	27.5 ± 1.0	22 ± 1.8	1.3 ± 0.1	73.8 ± 2.9	15.2 ± 1.5	2 ± 0.4	10 ± 1.2

^**a **^Number of amino acid residues

^**b **^pI, Theoretical isoelectric point

^**c **^A, Number of acidic residues

^**d **^B, Number of basic residues

^**e **^A/B, Ratio of acidic to basic residues

^**f **^AI, Aliphatic index

^**g **^Number of residues in the proteins

When compared to the Basal group, Epds from the other three groups show an increase in size. This increase is particularly evident in the MERPs with an average increase in size of 25 residues (or about 13%) over the Basal group. Our sequence comparisons show that it is in terms of the amino acid composition that the groups show highly significant differences. The predicted isoelectric point of the Basal group varies from 5 to 6.45. However, the FishBrain Epds show an acidic isoelectric point of around 5.1, while the isoelectric point of MERPs and FishTj Epds is around 6.5. The different isoelectric points are mainly due to a larger number of acidic residues and a decrease in basic residues found in FishBrain Epds, making the ratio of acidic to basic residues almost double that of other Epds.

Other differences among the Epd groups are noticeable. FishTj Epds have a significantly higher aliphatic index than the other groups. However, at the amino acid level, it is surprising that the number of Phe residues is almost halved in the MERPs in comparison to other groups. Similarly, the number of Trp residues in the molecules shows significant differences among the groups. The Basal species and the FishBrain molecules have an average of 2 Trps in their sequences, which increases to 3.2 in FishTj and to 6.6 in MERPs; this latter change is astonishing given that Trp is the largest and rarest amino acid. A similar trend is observed with the number of Pro residues, although the number only increases 50% between the Basal group and the MERPs. An additional comparative analysis of the predicted amino acid modifications in Epd proteins, including N-myristoylation, N-glycosylation and phosphorylation sites is available [see additional file 2: Additional_Text].

**Figure 5:**
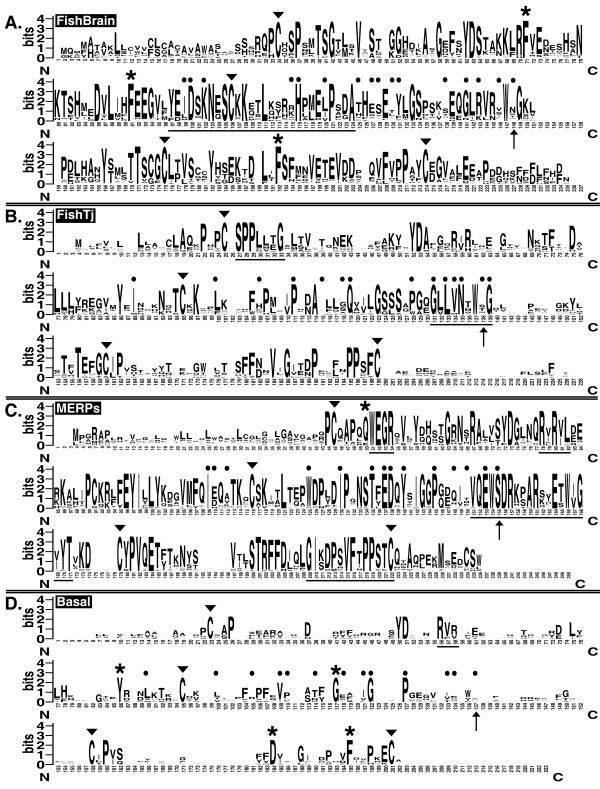
**Comparative analysis of the four ependymin protein family groups**. WebLogos were created from alignments that only included the sequences belonging to each ependymin group: **(A) **FishBrain – 33 sequences. **(B) **FishTj – 12 sequences. **(C) **MERPs – 20 sequences. **(D) **Basal – 8 sequences. For this analysis the Shark_Squa sequence was included in neither the FishBrain nor FishTj group. Underlined are the characteristic amino acid stretches for each group as described in the main text. Arrow heads and asterisks indicate diagnostic residues as in Figure 1. Functional divergence-related sites are labeled with a black circle above the letter. The rate-shifted site 161 used as example in the text is additionally labeled with an arrow. Regions of the alignments where gaps were introduced in most of the sequences are presented in the WebLogos as empty sites.

Apart from the amino acid residues that characterize the protein family (Figure 1); there are several amino acid features that typify each one Epd subgroup. These group-specific signatures were revealed using sequence logos generated from sub-alignments containing only the members of each group (Figure 5) [see additional file 2: Additional_Text]. This analysis clearly shows a divergent pattern of amino acid usage and conservation among the Epd family subgroups. This pattern suggests differences in selective constraints, likely arising from divergence in structural and functional aspects of the proteins phenotype.

### Analysis of selective pressures acting over the ependymin protein family

The fact that some Basal group sequences were found in protostomes (mollusks), lead us to suggest that this group represents the evolutionary origin of the Epd molecules. Alternatively, it may indicate that the Epd protein evolved in an ancestor of the protostomes and deuterostomes, but expanded into a gene family only within the deuterostomes. Under either scenario, the MERPs, FishBrain and FishTj groups represent the more derived members of the Epd protein family.

Different selective pressures acting over each Epd paralog group could have favored fixation of different sequences in each metazoan genome. To investigate the divergence of Epd groups, we used the rate of nonsynonymous (d_N_) and synonymous (d_S_) nucleotide substitution ratio (*ω*), as implemented in codon models of molecular evolution [47,48]. In this statistical approach, an *ω *< 1 indicates the action of purifying selection (i.e., a selective constraint against mutations that negatively impact the function of the protein); *ω *= 1 is consistent with neutral evolution, and *ω *> 1 indicates positive Darwinian selection (i.e., favoring the fixation of beneficial amino acid changes) [49].

We used the likelihood ratio test (LRT) statistic to determine if the selective pressure is significantly different between postduplication (PD) and postspeciation (PS) branches in the Epd phylogeny (Figure 4). We fitted two different PD-PS models to our data (see Methods) and contrasted each PD-PS model against a one-ratio model (M0) that assumed that PD branches as well as PS branches were subjected to the same selective pressure. The LRTs gave significantly higher support (*p *≤ 0.0003) to all the tested PD-PS models over the M0 model [see additional file 5: Table_S4]. Estimates of PD-PS model parameters suggest that (i) just after the duplication events, the fixation of amino acid changes increased in PD branches, and (ii) the rate of amino acid evolution decreased in PS branches, presumably due to more stringent levels of purifying selection. For instance, estimates under the Mps_1 _model were: *ω*_(PD) _= 1.139, *ω*_(PS) _= 0.161 and background *ω*_b _ratio = 0.056. In both tested PD-PS models d_N _values averaged 0.089 and d_S _values averaged 0.741.

Furthermore, our branch based analyses suggest that each Epd paralog group has been subjected to a different selective pressure during their natural history. The paralog models (Mp_1_, Mp_2_, and Mp_3_; see Methods) that allow for paralog-specific differences in selection pressure provided a significantly better explanation of the data (p < 0.0001, [see additional file 5: Table_S4]) than did the one-ratio model (M0) assuming no differences in selection pressure among Epd paralog groups.

Since each Epd group has particular amino acid features that differentiate it from other paralogs (Figure 5); we also expected the selective pressure to vary among sites and among Epd paralog groups. Therefore, we applied site-models of codon evolution to evaluate this scenario using each of the Epd groups (FishBrain, FishTj, MERPs and Basal) as separate data sets. When we applied the one-ratio site-model (M0) to each sub-dataset, the estimates of the *ω *ratio averaged over all sites for each paralog group (i.e., *ω*_MERPs _= 0.115, *ω*_FishBrain _= 0.182, *ω*_FishTj _= 0.195, *ω*_Basal _= 0.051) were all consistent with the *ω *estimates previously obtained under the Mp1 Paralog model [see additional file 5: Table_S4]. However, a LRT contrasting these separate analyses under M0 to M3, which allows among-site variation in the selective pressure, revealed significant heterogeneity in selective pressures within each member of the Epd family (*p *< 0.0001, [see additional file 5: Table_S4]). We note that this is not an unexpected result, as genes encoding functional protein products typically exhibit significant variation in selection pressure among sites. In addition, all Epd paralogs had a class of sites subjected to a rather strong purifying selection (*ω*_o _ranging form 0.003 to 0.014) but the fraction of such sites varies widely among paralogs (from 10% to 30%, [see additional file 5: Table_S4]).

Signature residues for the entire Epd family (Figure 1), such as the four strictly conserved Cys, are predicted to be critical for the common biological function of all Epd genes. In this context, we expected such residues to be localized in codon sites subjected to very strong purifying selection against nonsynonymous changes. When we applied the codon site models to the data set comprised of 70 Epd proteins (see Methods), we found that a LRT contrasting the site-models M0 and M3 was highly significant (*p *< 0.0001, [see additional file 5: Table_S4]), providing support for considerable variability in the selective pressures acting within the member genes of the Epd family. When we plotted the approximate posterior mean of the *ω *ratio at each codon site of the whole Epd protein family (Figure 1), the amino acids present in the most evolutionarily constrained positions with a *ω *≤ 0.04 were: C^45^, P^48^, G^53^, Y^73^, D^74^, E^98^, I^100^, F^109^, T^115^, C^118^, K^120^, P^134^, S^137^, G^146^, W^160^, C^188^, F^226^, P^229 ^and C^232^. For all these residues except for E^98^, the finding of strong evolutionary constraint is corroborated by our previous comparative analysis of sequence logos (Figure 1 and Figure 5). The E^98 ^site appears to be evolutionarily selected for acid residues in the more derived members of the Epd protein family; since this site is occupied by a Glu in all the members of the MERPs group, by an Asp in all the members of the FishBrain and FishTj groups. But in the Basal group this position is occupied by the aliphatic amino acid Leu.

The analysis of selective pressure acting in average over all sites of the entire data set and sub-datasets [see additional file 5: Table_S4], suggest that purifying selection (with among-site variability) has been the main influence on the evolution of the entire Epd protein family (*ω*_0 _= 0.1553) and their sub-groups (*ω*_MERPs _= 0.115, *ω*_FishBrain _= 0.182, *ω*_FishTj _= 0.195, *ω*_Basal _= 0.051). Moreover, none of the site-specific codon models (suitable for detecting adaptive evolution) implemented to the datasets, suggested the action of positive selection when the appropriate LRTs were performed (M1a vs. M2a, M7 vs. M8. data not shown). However, these site models averaged the selective pressure over all sites of the whole phylogeny or subgroups, and might have failed to detect short episodes of positive selection taking place over a few amino acid sites after a duplication event. Therefore, we implemented the branch-site models A and B [50-52] to detect if positive selection was driving the evolution of some sites along specific branches of the Epd phylogeny. These models let the *ω *ratio vary among sites and among lineages. We performed the Test 2 or "branch-site test of positive selection" [51,52] (see Methods) contrasting the model A against itself with *ω*_2 _fixed to 1 for each PD branch as defined in Figure 4 (FishBrain, FishTj, or MERPs). We found evidence of episodic adaptive evolution acting along the MERPs branch (*p *< 0.0001; [see additional file 5: Table_S4]). Five sites (86, 128, 196, 224 and 231 -numbered according to the WebLogo on Figure 1) had a high posterior probability (> 0.95) of being positively selected considering the Bayes Empirical Bayes (BEB) method as implemented on model A [51]. The site 86 is occupied in the MERPs group by basic amino acids with positively charged side chains (Arg, Lys, or His). In the FishBrain group this position is occupied only by polar amino acids being the more common Asn, Asp and Ser. Quite the opposite is observed for this position in the FishTj group, in which this site is occupied only by hydrophobic amino acids. However, in the Basal group this site is very variable and can be occupied by either polar or hydrophobic amino acids. The site 128 is strictly occupied by the aromatic amino acid Trp in all members of the MERPs group; it is preferentially occupied by aromatic residues (Phe, Tyr or Trp) in the FishTj and Basal groups. In contrast, in the FishBrain group this site is highly variable and usually occupied by Arg and Lys. The same analysis can be done for the sites 196, 224 and 231 that are preferentially occupied in the MERPs group by the hydroxylic amino acids Thr (sites 196 and 231) and Ser (site 224); while these sites are highly variable in the other paralog groups, being occupied mostly by non-hydroxylic polar residues.

Along the FishTj branch, we also obtained significant evidence for positive selection according to the Test 2 (*p *= 0.0233; [see additional file 5: Table_S4]), but only the amino acid site 144 was predicted with the BEB method under Model A. In the FishTj group, this site is occupied by an aliphatic amino acid (Val or Ile). In contrast, in the FishBrain group an aromatic residue (either Tyr or Phe) is usually present, except in the Fugu_Brain and the Tetraod_Br sequences that have a Ser. Interestingly, in the MERPs group, all the mammalian sequences have a Ser at position 144, but all other taxa within this group (i.e., fishes, amphibians, birds and echinoderms) have aromatic residues. Position 144 is highly variable in the Basal group and shows no clear pattern of amino acid replacement.

In contrast to the MERPs and FishTj groups, the evidence for positive selection affecting the FishBrain lineage was not as clear (*p *= 0.0747; [see additional file 5: Table_S4]), and can be considered only to be marginal support for adaptive selection or relaxed selective constraints at three sites (51, 126 and 186). In the FishBrain group, the residue in position 51 is almost always occupied by the hydroxylic amino acid Thr, except in the sequences Rhamphicht and 2Carassius which have Ile in this site. This position is occupied by non-polar amino acids in all other groups, and is always Trp in the MERPs group. The site 126 in the FishBrain group can be occupied by polar (Tyr, Ser or Cys) and non-polar amino acids (Phe); but in the MERPs group this position is only occupied by polar amino acids such as Gln, and specially the acidic amino acids Glu and Asp. The amino acid composition of this site for the FishTj and Basal groups is highly variable, without a clear pattern. Position 186 is one of four contiguous amino acids conserved in all FishBrain and FishTj groups (but not in the fish sequences belonging to the MERPs group), and in the sequences Sea_cucumb and Ciona_Tun2. These amino acids appear to be specifically inserted in FishBrain and FishTj sequences and later on by convergence, the sequences from echinoderms and one of the *Ciona epd *genes might have acquired it. Alternatively, these four amino acids could have been lost specifically in the ancestral MERP sequence and also in certain Basal groups, with exception of the echinoderms and one of the *Ciona *sequences.

Almost all the available sequences from the FishBrain group have a Cys in position 186, which is one amino acid before the third common Cys (C^188^). Only in Percomorpha and Salmoniformes is this Cys substituted by Gly. These cysteines, which are very close together in the Epd linear structure, are predicted by the Disulfind server [30,35] to form disulfide bonds. The consistent occurrence of Gly, a small amino acid with a very high conformational flexibility, between the C^186 ^and C^188 ^would permit a disulfide bridge between them, since Gly does not present a steric obstacle. Alternatively, although with a lower confidence of connectivity, C^186 ^may participate in shuffling reactions forming potentially an array of disulfide intermediate species as result of its binding with other cysteines [53]. Only when the crystallographic structure of proteins belonging to the different Epd protein subgroups is obtained and careful functional assays are performed, we will be able to test if the different possible transition stages of disulfide bonding are having an impact on the functional divergence among Epds paralogs.

### Detection of functional divergence among ependymin paralogs

Detectable differences in the site-rate of amino acid replacement between Epd paralog groups can give us an idea of the grade of functional divergence generated since the duplicated genes diverged, splitting ancestral functions or generating new ones, and consequently succeeded avoiding pseudogenization. The coefficient of evolutionary functional divergence (*θ*) obtained for each Epd paralog pair comparison was significantly greater than zero (Table 2), indicating that there is significant heterogeneity in the amino acid site-specific rate of evolution among Epd paralogs. This result further supports the estimates obtained at the codon level by applying Paralog models and site-models to separate datasets of the paralogs (see previous section). That is, that each Epd group has been subjected to different functional constraints in specific amino acid sites and therefore, functional divergence among them can be inferred.

**Table 2:** Maximum likelihood estimates of the coefficient of functional divergence (θ) from pairwise comparisons between ependymin groups

**Comparison**^**a**^	***θ *± SE**^**b**^	**LRT**^**c**^	**α**^**d**^
FishBrain/MERPs	0.85 ± 0.12	52.20	1.12
FishTj/Basal	0.81 ± 0.15	28.60	1.96
MERPs/Basal	0.70 ± 0.21	11.27	1.20
FishBrain/Basal	0.63 ± 0.13	24.25	2.31
FishTj/MERPs	0.52 ± 0.09	30.02	0.90
FishBrain/FishTj	0.35 ± 0.09	14.13	1.21

^**a **^The number of sequences considered in each gene cluster for the program DIVERGE v1.04 [80, 81] was: FishBrain (32), FishTj (12), MERPs (17), and Basal (8). The Shark ependymin was not included neither in the FishBrain nor FishTj gene clusters.

^**b **^SE, standard error.

^**c **^LRT, likelihood ratio test.

^**d **^α, Gamma shape parameter of rate variation among sites.

The six possible pairwise comparisons among Epd paralog groups were performed (Table 2). The comparison between the FishBrain and MERPs groups showed the highest value for *θ *(0.85 ± 0.12), suggesting that these two groups have diverged considerably more at the functional level. This estimate is supported by the fact that the expression pattern of these proteins is the more dissimilar, being the proteins belonging to the FishBrain group expressed exclusively in brain tissue [13-15], and the ones from the MERPs group expressed in several tissues including brain [7,12]. High *θ *values are also found for each one of the pairwise comparisons that can be done against the Basal group, suggesting that if the proteins belonging to the Basal group are in fact the root of the *epd *gene tree, the derived groups have significantly diverged functionally from the ancestral gene function, expression pattern or both. This measure of functional divergence among the groups (FishBrain, FishTj and MERPs) that were contrasted with the Basal group was not equidistant: the predicted functional divergence between the FishTj group and the Basal group (*θ *= 0.81 ± 0.15) is higher than that for FishBrain vs. Basal (*θ *= 0.63 ± 0.13) or MERPs vs. Basal (*θ *= 0.70 ± 0.21). As could be expected from the gene tree (Figure 4), the smallest *θ *value (being however also significantly greater than zero) was obtained for the FishBrain vs. FishTj comparison (*θ *= 0.35 ± 0.09). The inferred phylogenetic gene tree suggests us that the duplication that gave rise to the FishBrain and FishTj paralog *epd *genes was posterior to the appearance of the MERPs group. Thus, the FishTj and FishBrain shared more time together and although the FishBrain group already has a very restricted pattern of expression that contrasts with the ample array of tissues from which FishTj genes have been isolated, we might suppose that some functional overlap may remain. Of course, it will be interesting to corroborate this assumption with experimental data where knocking out one of the genes is compensated (at least partially) by the other. Interestingly, the estimated coefficient of functional divergence between the FishTj and the MERPs group (*θ *= 0.52 ± 0.09) is not as high as we could suppose from the gene tree, although this would suggest different functional roles it can not exclude that some of these functions overlap.

**Figure 6:**
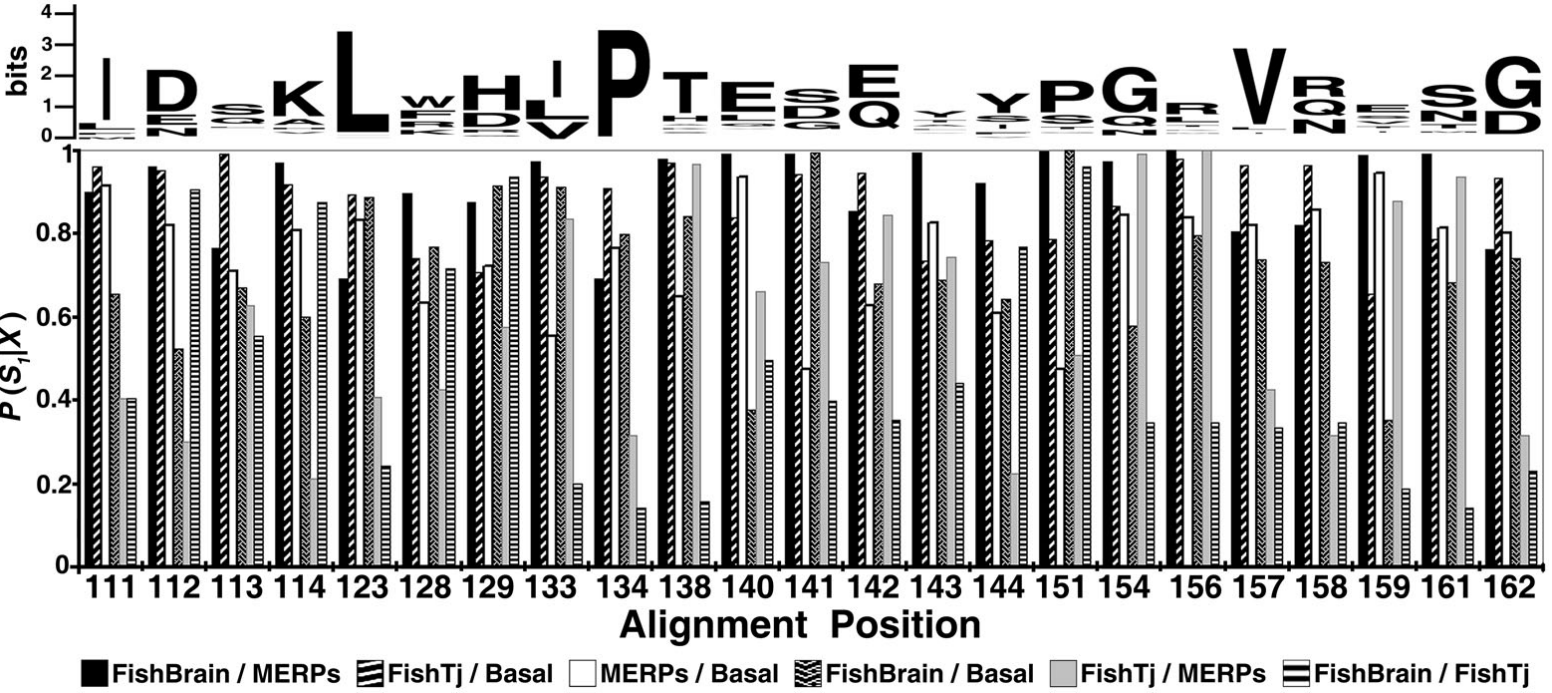
**Prediction of functional divergence-related sites among ependymin protein groups**. Site-specific profile showing the amino acids predicted to have a posterior probability [*P*(*S*_*1*_|*X*) > 0.90] of being functional divergence-related sites in any of the pairwise comparisons between the ependymin groups. The corresponding amino acid residue for each site as calculated for sequence logo on Figure 1 is shown above the plot.

Posterior Bayesian analysis predicted several amino acid positions that may account for the inferred functional divergence among the Epd paralog groups (Figure 6). Even though, sites with an estimated posterior probability (PP) < 0.8 have been experimentally proved to be important for the observed functional divergence between the two major Caspase subfamilies [54]; the cutoff value for residue selection is an empirical decision and is expected to depend on the intrinsic properties of the protein family being analyzed. Thus, while in [54] they obtained 21 candidate functional divergence-related sites using 0.61 as cutoff value, the same cutoff value predicts 53 sites for the Epd data. Since no 3D structure of any Epd protein is yet available, we lack a way to verify where these sites would be located nor how the rate-shift in these sites contributed to structural and functional divergence among the Epd paralogs. Nonetheless, we can further narrow our criteria for site prediction expecting that in the case of the Epd data, sites predicted with a more stringent PP (i.e., > 0.9) be in fact functional divergence-related sites that can serve as a discrete starting point for future functional characterization of the Epd proteins. Twenty-three rate-shifted amino acid sites (8.8% of total sites) were predicted with a PP > 0.9 of being functional divergence-related sites for any of the six possible pairwise comparisons among Epd paralog groups (Figure 6). The rate-shifted site 161 (the amino acid after the common Trp of the Epds -see Figure 1), for example, is predicted with a PP = 0.99 to be able to functionally distinguish the members of the FishBrain from the members of the MERPs group, with a PP = 0.93 to distinguish the FishTj from the MERPs group, and with intermediate PPs to distinguish FishBrain from Basal (PP = 0.68), FishTj from Basal (PP = 0.79) and MERPs from Basal (PP = 0.82); but this same site only has a PP = 0.14 to discriminate FishBrain from FishTj proteins. When this site was localized in the subgroups alignments (site indicated by an arrow in the Figure 5), it is clear that being highly variable, the site is not useful for discriminating between FishBrain and FishTj proteins. In contrast, the MERPs Epd proteins have a Ser fixed in this position which is within the stretches of amino acids diagnostic of this group (QEW**S**DR--aR--E-WXGxyT, underlined in the MERPs WebLogo, Figure 5C). In addition, in the Basal group site 161 is occupied with almost equal occurrence by Ser, Asn, His, Tyr, or Arg. Consequently, once the 3D structure of Epd proteins from the differences subgroups are available, and site-directed functional experiments can be done, we expect to be able to corroborate that the rate-shifted site 161 is somehow important to the particular function of the MERPs Epds.

A similar analysis can be performed for all the other 22 rate-shifted predicted sites. It is worth noting that sites 128 and 144, predicted to be under positive selection in the MERPs and FishTj lineages respectively by using branch-site codon models (see previous section), were also predicted to be functional divergence-related sites using this very different approach. As previously noted, the site 128 is preferentially occupied by aromatic residues in the FishTj, Basal and MERPs groups, being always occupied by a Trp in the latter; but in the FishBrain group this site is very variable. This explains why this site has a PP = 0.90 of being related with the type 1 functional divergence between the MERPs and FishBrain groups, and has intermediate PPs for the other possible comparisons: FishBrain vs. FishTj (PP = 0.72), FishBrain vs. Basal (PP = 0.77), FishTj vs. Basal (PP = 0.74), MERPs vs. Basal (PP = 0.63) and FishTj vs. MERPs (PP = 0.43). It is also noteworthy that the presence of aromatic amino acids at this site can distinguish the Epd proteins that are exclusively expressed in the brain, from the proteins that have a wider expression pattern. Additionally, the presence of a Trp at this site appears to be important for the function of the Epds belonging to the MERPs group as suggested by the convergent results obtained using codon models and the search for type 1 functional divergence related sites (see Methods).

Position 144 was predicted to be under positive selection and additionally to be related to functional divergence. This site has a PP = 0.92 for discriminating between the FishBrain and the MERPs groups and with less confidence can distinguish between other groups: FishTj vs. Basal (PP = 0.78), FishTj vs. Brain (PP = 0.77), FishBrain vs. Basal (PP = 0.64), MERPs vs. Basal (PP = 0.61) and FishTj vs. MERPs (PP = 0.22). Contrary to what occurs with site 128, the relationship of aromatic amino acids and tissue specific expression appears to be inverse in site 144. Thus, the preferential occurrence of aromatic amino acids in the FishBrain group (particularly Tyr) appears to be important for the functional divergence of FishBrain group as compared with the others.

## Conclusion

The Epd protein family is not deuterostome-specific as previously thought. We provide evidence that Epds are also present in protostomes. Our results suggest that fish-specific gene and/or genome duplications triggered diversification of the Epd protein family into four groups. Two of these groups (FishBrain and FishTj) are unique to fishes; a third appears to be deuterostome-specific (MERPs), and the fourth (Basal) has been only found in invertebrate deuterostomes and protostomes. Statistical analyses of selective pressures indicate that episodic positive selection and relaxed selective constraints have shaped the evolution of the Epd proteins. However, purifying selection (with among-site variability) appears to be the main influence on the evolution of each subgroup within the family. We detected functional divergence among the Epd groups and amino acid sites that account for it. Taken as a whole, our phylogenetic results and the tissue-specific patterns of expression of the different Epd proteins, suggest that this protein family is a suitable target to experimentally test subfunctionalization in gene copies resulting from duplication events [55,56]. We reiterate that protein structural information and more experimental data including functional knocking out assays remains to be obtained from representative members of the four Epd protein groups described here. Thus, we hope that our findings encourage and serve as useful guidelines to further research on how evolution shaped the Epd protein family, their conserved and divergent gene functions.

## Methods

### Gathering of sequences

Thirty five sequences previously used in phylogenetic analyses of the Epd protein family [6,7,12] were used in the present study as starting material to search several data bases for additional family members. The taxonomic group, working names, species names, accession numbers and bibliographic information of these sequences are provided [see additional file 3: Table_S2]. [See additional file 2: Additional_Text for a detailed description of the methods used for searching and identifying new Epds, including the tests performed to substantiate the placement of each one into the Epd protein family].

### Virtual mapping

For genome searches and cross mapping of *epd *genes we used publicly available databases. This included the Ensembl release v40 [57] and the specific genome browsers for each sequenced species such as Genome Browser at Genoscope [58] for *Tetraodon *and Genome Browser at University of Tokyo [59] for medaka. To find exact matches to a chromosome location, contig or scaffold we used the Sequence Search and Alignment by Hashing Algorithm (SSAHA tool in Ensembl) [60] to align DNA sequences to the current assembly within Ensembl. Once two or more paralog *epd *genes from the same species were found, a ClustalW alignment was performed, and the percentage of similarity among them was determined.

### Sequence alignments

All the Epd sequences were aligned with the software ClustalX v.1.81 [61] using the BLOSUM30 matrix. GeneDoc v.2.6.002 was used for manual improvement of the alignment by eye. This led to an alignment of 74 Epd domain-containing proteins (35 previously known and 39 new sequences) having 261 amino acid positions. Sequence logos for the protein alignments (whole dataset and subsets) were created online using WebLogo Version 2.8.2 [33,34]. All the multiple sequence alignments are available upon request.

### Phylogenetic analysis

We carried out phylogenetic inference on the full alignment of 74 sequences. Protein trees were built using Maximum-likelihood (ML), Bayesian inference, Neighbor-Joining (NJ), and Maximum Parsimony (MP). The ML optimality criterion as implemented in PHYML v2.4.4 [62] was used applying the WAG amino acid substitution matrix, empirical model for globular proteins [63], accounting for site-to-site rate variation using a discrete gamma distribution with four rate categories and invariable sites; the proportion of invariable sites was estimated from the data and support for individual branches was inferred by bootstrap analyses (100 replicates). For Bayesian inference we used MrBayes v3.0b4 [64] making five independent runs using the same settings: starting with a random tree, using mixed models of amino acid substitutions, generating trees for 5 million generations with sampling every 100 generations, and with four chains (three with the heating parameter set to 0.2). The first 1,250,000 generations (12,500 trees) were discarded from every run and the remaining trees were concatenated. The remaining 187,500 trees were used to compute the final (consensus) tree, and to determine the posterior probabilities at the different nodes. The convergence of the five independent runs was calculated and confirmed as described in [65]. NJ and MP trees were built using PAUP* v.4.0b10 for Unix [66]. The NJ tree [67] was constructed based on mean character differences (distance) and bootstrap support values were determined based on 10,000 neighbor joining replicates. The MP tree [68] was obtained using the heuristic search method. All the 261 amino acid characters had equal weight and 239 of them were parsimony-informative. The MP tree was built via random stepwise addition of the sequences and the tree-bisection-reconnection (TBR) branch-swapping algorithm (NR = 10) with 100 bootstrap replicates.

### Tests of tree topologies

The inferred gene tree topology was tested against the expected species tree [69] for the cases in which they were not coincident. For a given comparison between the gene tree topology and the alternative topology derived from the expected species relationships, the Kishino-Hasegawa (KH) [43] and Shimodaira-Hasegawa (SH) [42] tests were performed in both cases using a time-saving approximation, the RELL method with 10,000 replicates of nonparametric bootstrapping [43]. Two trees were compared each time; the ML tree in Figure 4 and a tree modified using the tree editor of TreeView v.1.6.6 [70] to relocate misplaced taxa in agreement with the species phylogeny [71]. Tests were carried out as implemented in Codeml (PAML package V.3.14b) [72,73] by using unrooted trees, and assuming the empirical model WAG.

### Statistical analyses of selective pressure

To study the selective pressure on the Epd domain-containing proteins, we excluded the Paracheiro, Sea_urchin, Chicken_Gg, and Epdr_Chimp sequences to avoid small sectors of uncertainty due to the presence of scattered Ns in their nucleotide sequences. These nucleotide uncertainties, however, do not affect their amino acid translation, allowing their inclusion on the amino acid-based analyses described above. The nucleotide sequences for the remaining 70 lineages were aligned according to their translated protein sequence using CodonAlign 2.0. With the codon alignment and the corresponding unrooted ML protein tree, we implemented several Markov models of codon evolution (which describe the substitution process between 61 of the 64 codons) to statistically estimate the selective pressures acting over all codon sites, specific branches, and sites within given branches of the Epd protein family phylogeny. In these analyses, maximum likelihood estimates of the selective pressure (*ω*) were obtained as the nonsynonymous (d_N_) and synonymous (d_S_) nucleotide substitution rate ratio (d_N_/d_S_) with a d_N_/d_S _(*ω*) = 1 meaning neutral evolution, *ω *< 1 purifying selection, and *ω *> 1 diversifying positive selection [49]. The first and simplest model considered was the M0 (one-ratio model), which assumes the same *ω *ratio for all branches in the phylogeny and all codon sites in the alignment. Branch models allow for different values of *ω *along branches in the tree [74,75]; we applied them to analyze the outcome of the putative duplication events that occurred during the natural history of the Epd protein family. We implemented the PD-PS model [71] that estimates different *ω *ratios for postduplication (PD) and postspeciation (PS) branches in a tree and allows to test if the rate of amino acid replacement along PD branches is significantly higher compared to PS branches. Specifically, we were interested if fixation of nonsynonymous amino acid changes has been favored by positive selection following a duplication event (PD branches), and then decreased once a new function had evolved due to the dominance of purifying selection (PS branches) [76,77]. We fitted two PD-PS models to our data (Figure 4). The first model (Mps_1_) assigned one *ω *ratio to the PD branches in the tree [*ω*_MERPs(PD)_, *ω*_FishBrain(PD)_, and *ω*_FishTj(PD)_], a second *ω *to their corresponding PS branches [*ω*_MERPs(PS)_, *ω*_FishBrain(PS)_, and *ω*_FishTj(PS)_], and a third *ω *to all remaining branches (background ratio *ω*_b_). The second PD-PS model (Mps_2_) assigned one *ω *to the PD branches immediately postdating the putative gene duplication event D_2 _[*ω*_FishBrain(PD) _and *ω*_FishTj(PD)_], a second *ω *to their corresponding PS branches [*ω*_FishBrain(PS) _and *ω*_FishTj(PS)_], and a third *ω*_b_, for all other branches. A likelihood ratio test (LRT) was done to compare the M0 one-ratio model [*ω*_(PD) _= *ω*_(PS)_] with each one of the PD-PS models [i.e., *ω*_(PD) _≠ *ω*_(PS) _in Mps_1 _and Mps_2_]. The LRT statistic was calculated as twice the difference between the log likelihood scores of the two nested models, and was compared to the chi-square distribution with degrees of freedom (df) equal to the difference in number of free parameters between the two models [see additional file 5: Table_S4].

We also implemented the Paralog model [71]; which allows testing for divergent selection pressures among paralogs following a gene duplication event. Hypothetically, if this subfunctionalization [21] involved both the regulatory sequences and the protein coding sequences, there could be differential loss of selection pressure on different parts of the protein detectable with a Paralog branch model [71]. We fitted three Paralog models to our data. The first model (Mp_1_) estimated one *ω *ratio for each of the paralog clades derived from the Basal Epd group (*ω*_MERPs_, *ω*_FishBrain _and *ω*_FishTj_) and one *ω*_b _background ratio. The second Paralog model (Mp_2_) assumed one *ω *for each of the paralog piscine clades originating after the D_2 _duplication (*ω*_FishBrain _and *ω*_FishTj_), and another *ω*_b _for all other branches. The third Paralog model (Mp3) estimated one *ω*_b _background ratio, one *ω *for the MERPS paralog clade (*ω*_MERPs_), and one (*ω*_Non-MERPs_) for all others. A LRT comparing the M0 one-ratio model (*ω*_MERPs _= *ω*_FishBrain _= *ω*_FishTj_) with each one of the Paralog models (e.g., Mp1: *ω*_MERPs _≠ *ω*_FishBrain _≠ *ω*_FishTj_) was then performed to test for significant differences in selective pressure among Epd paralogs [see additional file 5: Table_S4].

We also implemented site-based models of codon evolution that allowed the *ω *ratio to vary among sites (among codons or amino acids in the protein) [51,78,79]. A model (M3) that allows for three discrete classes (k = 3) of *ω *within the gene was contrasted with a LRT against a one-ratio model (M0) in which the *ω *ratio averaged over all gene sites. Other LRTs contrasted additional site-models evaluated to detect positive selection acting in average over all sites in the phylogeny: M1a (NearlyNeutral) against M2a (PositiveSelection), and M7 (beta) against M8 (beta&*ω*). Given that these site models might not detect positive selection affecting only a few sites along a few lineages after a duplication event, we also implemented the branch-site models A and B which let the *ω *ratio vary both among sites and among lineages [50-52]. The Models A and B had four *ω *site classes with three estimates of the *ω *ratio: site class 0 included strongly conserved codon sites in all branches (0 <*ω*_0 _< 1); site class 1 included neutrally evolving sites across all branches (*ω*_1 _= 1); and site classes 2a and 2b included those sites that were either conserved (0 <*ω*_0 _< 1) or neutral (*ω*_1 _= 1) on background branches but with a *ω*_2 _> 1 on the foreground branch of interest (i.e., PD branches in the Epd tree topology). Model A estimated *ω*_0 _(0 <*ω*_0 _<1) from the data and fixed *ω*_1 _= 1, while model B estimated the *ω*_0 _and *ω*_1 _parameters from the data. Based on these models we were able to conduct the following LRTs: (i) model B versus a discrete model M3 with (K = 2) site classes, (ii) model A versus M1a [Test 1], and (iii) model A with *ω*_2 _as a free parameter versus model A with *ω*_2 _fixed at 1. This test [Test 2] is known as "the branch-site test of positive selection"; see [51,52] for a detailed description of these latter two tests.

These analyses of selective pressure were performed by using the Codeml program of the PAML package V.3.14b [72,73]. All tree topologies were unrooted. For each model, convergence of the ML estimates was always checked by running all analyses three times with different starting *ω *values. We also used alternatively the CodonFreq = 2 (F3 × 4) or CodonFreq = 3 (F61) to run the analyses. Similar LRTs results were obtained when either CodonFreq option was used. Given an *ω *> 1 and a significant LRT under model A, the Bayes Empirical Bayes (BEB) approach was used to infer amino acid sites under positive selection [51]. Bayesian and maximum likelihood analyses were run on the Espresso Linux cluster at the High Performance Computer Facility of the University of Puerto Rico (HPCf-UPR).

### Test of functional divergence

We estimated the functional divergence among the four proposed paralog groups (i.e., Basal, MERPs, FishBrain, and FishTj). The ML protein alignment and topology showing the relationship among 70 Epd domain-containing proteins were used as input for the program DIVERGE v1.04 for calculating the coefficient of functional divergence (*θ*) between pairs of paralog groups [80]. The parameter *θ *is a maximum likelihood estimator of type 1 functional divergence between two members of a protein family and is based on the detection of altered rates of amino acid replacements for a given position between the two paralog groups. That is, when an amino acid configuration is very conserved in one of the paralogs and is highly variable or fast evolving in the other, it implies that this site-specific rate difference originated because the paralogs experienced different functional constraints at these residues [54]. A LRT was then done to test the null hypothesis *θ *= 0 against the alternate hypothesis of *θ *> 0. If the estimated *θ *was significantly greater than zero, functional divergence between the paralog pair was highly supported. A Bayesian approach was then followed to statistically predict which sites were likely to be responsible for the type 1 functional differences found between the paralogs [81]. For our analyses, those positions with posterior probabilities > 0.9 in the site-specific profile for each pairwise comparison were considered to be potential functional divergence-related amino acid sites.

## List of abbreviations

Epd, Ependymin; Epdr, Ependymin Related Protein; ESTs, Expressed Sequence Tags, SSAHA, Sequence Search and Alignment by Hashing Algorithm; CDD, Conserved Protein Domains; LG, Linkage group; NJ, Neighbor-Joining; MP, Maximum Parsimony; ML, Maximum Likelihood; LRT, Likelihood Ratio Test; BEB, Bayes Empirical Bayes; PP, Posterior probability.

## Authors' contributions

ECSC conceived, designed and carried out the study, performed statistical analyses, drafted the manuscript and prepared the figures. JEGA helped design the study, analyzing results and draft the manuscript. Both authors read and approved the final manuscript.

## Supplementary Material

Additional file 1 **Newly identified ependymin proteins**. In this table the taxonomic groups, working names, species names, accession numbers, tissue expression and bibliographic information (if available) are provided . In addition, the best BlastP e-value of each new ependymin sequence to a previously known member of this protein family is given . Click here for file

Additional file 2 **Supplemental methods & results and discussion**. This file includes: **(A) **– Detailed methods followed for searching and identifying new ependymins; **(B) **– Caveat about the mouse ependymin genes; **(C) **– Comparative analysis of the predicted amino acid modifications in ependymin proteins; **(D) **– Amino acid signatures that define each ependymin protein group; and **(E) **– References used in additional files. Click here for file

Additional file 3 **Formerly published ependymin proteins**. Ependymin sequences obtained from published work and used as starting material for our study. In this table are provided the taxonomic group, working names, species names, accession numbers, tissue expression and bibliographic information. Click here for file

Additional file 4 **Virtual mapping of ependymins**. The linkage group (LG) or chromosome (Cr) location to which each transcript was mapped is indicated. Also Ensembl, Genoscope, Genescan or Genewise identifiers for each sequence and the Unique Best Recipocal Hits (UBRHs) predicted for each of them by Reciprocal Blast Analysis are provided. In this table gene copies from the same organism are grouped. Click here for file

Additional file 5 **Statistical Analyses of Selective Pressure**. Parameter estimates of *ω *ratios, log-likelihood scores and LRT statistics for the ependymin data under different branch-, site-, and branch-site models of codon evolution. Click here for file
