# Supplementary material for: Molecular evolution of the ependymin protein family: a necessary update
Source: BMC Evol Biol. 2007 Feb 15;7:23. doi: 10.1186/1471-2148-7-23 (PMC1805737; doi:10.1186/1471-2148-7-23)
Supplement: Additional file 2 — Supplemental methods & results and discussion. This file includes: (A) – Detailed methods followed for searching and identifying new ependymins; (B) – Caveat about the mouse ependymin genes; (C) – Comparative analysis of the predicted amino acid modifications in ependymin proteins; (D) – Amino acid signatures that define each ependymin protein group; and (E) – References used in additional files. [file 1471-2148-7-23-S2.pdf]

Supplemental methods & results and discussion for:

Molecular Evolution of the Ependymin Protein Family: A Necessary Update

Edna C. Suárez-Castillo and José E. García-Arrarás

Department of Biology, University of Puerto Rico, Río Piedras Campus, Puerto Rico 00931

This file includes:

(A) – Detailed methods followed for searching and identifying new ependymins

(B) - Caveat about the mouse ependymin genes

(C) - Comparative analysis of the predicted amino acid modifications in ependymin proteins

(D) – Amino acid signatures that define each ependymin protein group

(E) – References used in additional files

Detailed methods followed for searching and identifying new ependymins

Public databases that were searched for previously unrecognized ependymin sequences included: GenBank [1], dbEST [2], Ensembl [3], Genoscope [4] and individual EST and genome projects such as for sea urchin the NCBI's Sea Urchin Genome Resources [5] and the Max Planck Institute Sea Urchin Gene Catalogue [6]; for medaka the Mbase [7], the UTGenome Browser [8] and the NBRP database [9]. Where available, we used the protein sequence translations associated with the original author's submission. Alternatively, when the submitting author's translation of the ESTs or genomic sequences was not available, the most probable open reading frame (as assessed by the BlastX searches with higher score) was determined using the Sixframe option on Biology Workbench 3.2 [10]. In cases where several very similar or overlapping DNA sequences were obtained (from redundant ESTs or several readings in genome projects) and a consensus sequence was not provided by the

submitting author, these sequences were used as input for the web-based contig assembly program CAP3 [11, 12]. In most cases, this contig assembly process had enough sensitivity to give DNA sequences that, once translated, produced sequences with a single non-fragmented open reading frame [13].

We used well-known ependymin sequences as anchors to retrieve gene sequences from public databases that were not cross-referenced with the ependymin gene family (often referenced as “unnamed protein product” or “hypothetical protein”). Specifically, these anchor sequences were used as queries in reverse position-specific iterated BLAST algorithm (PSI-BLAST) [14], tBlastn and/or tBlastx searches [15] against the databases of non-redundant (NR) sequences and ESTs (filtering human and mouse sequences). All the UBRH (Unique Best Reciprocal Hits) obtained by this reciprocal Blast analysis [16] were treated as putative orthologues, their sequences were further analyzed, and a decision was taken on whether a given sequence (complete mRNAs, ESTs, or predicted from a genome sequence) was reliable enough to be included in the main data set for phylogenetic inference. In addition, all the links provided in the Ensembl [17] homepage for each gene from a sequenced or in progress genome project in which a *bona fide* ependymin gene has been previously reported (i.e., zebrafish and human) were inspected searching for additional predicted homologues of ependymin sequences or UBRHs present in others Ensembl hosted genomes.

The InterPro database [18] and the NCBI’s Conserved Domain Database (CDD.v2.05) [19, 20] were used to scan the putative new sequences and substantiate their placement into the ependymin protein family**.** Detection of the ependymin domain in at least 105 consecutive amino acids was used as criterion for proper assignment of a given sequence to the protein family. The established sequence hallmarks of ependymin proteins [21-23] were searched for each sequence. The presence and localization of signal peptides was assessed with the web

based SignalP 3.0 Server [24]. The prediction of N-glycosylation sites was run in the NetNGlyc 1.0 Server [25]. Hydropathic profiles were obtained in ProtScale [26] by using normalized (from 0 to 1) Kyte-Doolittle scores [27] over a window length of 9. The prediction of cysteine bonding state and connectivity was run on the Disulfind server [28, 29]. Physical and chemical parameters for each protein sequence were computed using the ProtParam tool [30]. Other tools for protein sequence analysis were run on the PredictProtein server [31].

Caveat about the mouse ependymin genes

Two ependymin genes from mouse are described in the literature [22] and in GenBank, the *Epdr1_Mus* [GenBank:AY027861] and the *Epdr2_Mus* [GenBank:AF353717]. However, in Ensembl [3] the *Epdr2_Mus* is the only one characterized, mapped and cross-referenced to the other ependymin genes [see additional file 4: Table_S3 for detailed results from mapping]. We attempted to map the *Epdr1_Mus* gene to the mouse genome using its reported nucleotide sequence as input for the SSAHA tool [32], but it mapped to the same genome location of the *Epdr2_Mus* gene, although with a lower score. However, when we used the nucleotide sequence of *Epdr1_Mus* as input in SSAHA and mapped it against the human genome, we obtained a perfect match to exactly the same genome location of the *Epdr1_Homo* gene, obtaining similar high scores as when the *Epdr1_Homo* was used as input. This result questions the existence of two different ependymin genes in the mouse genome. The percentage of amino acid difference between Epdr1_Mus and Epdr2_Mus is only 6.25%, which is very similar to the percentage of difference between Epdr2_Mus and the human sequence Epdr1_Homo (5.80%). Particularly intriguing is the difference between Epdr1_Mus and Epdr1_Homo, which is only 0.45 % (only two amino acids are different); and at the nucleotide level the coding sequences of these two genes have 99.3% identity. Thus,

from the map data and the percentage of similarity, we suggest that *Epdr1_Mus* is actually product of human contamination of a PCR reaction made from a mouse C57BL/6 thymus cDNA library using primers from a human sequence [22].

Comparative analysis of the predicted amino acid modifications in ependymin proteins

Additional information has been obtained from predicted amino acid modifications, including N-myristoylation, N-glycosylation and phosphorylation sites. Most ependymin proteins have at least two putative glycosylation sites. Exceptions to this are Ciona_Tun2 with none and Fugu_Tj, Diplo_Tun and Aplysia with only one. The location of the putative glycosylation sites varies according to the groups: FishBrain ependymins show putative glycosylation sites at 69-77 and 92-101, while for the other groups the sites differ: MERPs (113-132 and 166-196) FishTj (64-69 and 89-132). Similarly, most ependymins have at least one predicted myristoylation site. Exceptions to this are Esox_luciu, Salmo_Bra, Xetr_Frog and Aplysia. However, the site of the predicted myristoylation varies. In FishBrain, 10 out of the 13 species have the site localized between residues 133 and 166. In MERPs, with the exception of Sea_cucumb and Danio_MERP, the myristoylation site is within the signal sequence. Finally, in FishTj, two sites can be predicted: One present in residues 62 or 63 and one between 126 and 128. The sole exception to these two sites is Salmo_IH where the predicted myristoylation sites are residues 130 and 201.

Except for the Basal group, all other ependymin groups show particular predicted phosphorylation sites. The Basal group is too variable to define a particular trend in these predictions. In the FishTj group, 7 out of 9 sequences show a predicted Protein kinase C (PKC) phosphorylation site between residues 72 and 92, while the remaining two show the site at positions closer to the carboxyl terminal (141 and 163). Six out of the 9 sequences

show a Tyr phosphorylation site between residues 43-57. All sequences have at least one predicted Casein kinase II (CK2) phosphorylation site between residues 147 and 194. MERPs also have at least one CK2 phosphorylation site 115-134, and all vertebrate species show a second putative site between residues 158-183. In addition, MERPs are characterized by at least 4 different predicted PKC phosphorylation sites, the most common residues being those around 57-62, 152, 156-158, 168-178 and 188-202. In contrast, most of the predicted PKC phosphorylation sites for FishBrain ependymins lie near the amino terminal of the molecule. However, only one of them, between residues 95 and 103, is found in the majority (10 out of 13) of the molecules. These same molecules show a predicted cAMP- and cGMP-dependent protein kinase phosphorylation site between residues 97 and 105. Finally, with the exception of Fugu_Brain, Tetraod_Br and Clupea which only show 2-3 predicted CK2 phosphorylation sites; most FishBrain ependymin sequences show at least five predicted sites. The most common positions for these sites are residues 66-77, 91-95, 126-130, 178-184, and 196-210.

Amino acid signatures that define each ependymin protein group

FishBrain Epds (Figure 5A) are characterized by having a conserved sequence around the second Cys (C^{106}) that starts with either a Phe or a Tyr: $[Y/F]^{97}$ZXZ-kNZSC-K--L----HXXZXP--A^{124}. This region is rich in acidic residues, 7-8 per sequence, with the most commonly acidic positions marked with Zs. Hydrophobic residues are common in positions labeled with Xs. In this notation, numbers refer to the appropriate WebLogo in Figure 5, hyphens mean any residue, and amino acids in lower case indicate that only one or two sequences in the group do not display the corresponding residue. FishBrain Epds also have characteristic Phe residues in their sequences. Three of these are found in specific sites: F^{71}

located eight residues down from the common Asp (D^{63}), F^{91} twenty more residues farther down, and F^{192} seventeen residues down from the third Cys (C^{175}).

The FishTj group (Figure 5B) can be characterized mainly by the S^{27}PP---G^{33} sequence found one amino acid downstream from the initial Cys (C^{25}). The second characteristic of this group is a stretch of amino acids G^{131}XLvN-W-G^{139} where W^{137} is the Trp common to all Epds and the X is either Leu or Val.

The first identifiable trait of the MERPs group (Figure 5C) is the sequence W^{51}EGR^{54} that can be found five amino acids after the first Cys (C^{45}). A Gln (Q^{50}) usually precedes this sequence except for Chicken_Gg and Fugu_MERP where a Glu has been substituted. The other sequence characteristic of the MERPs group is Q^{152}EWSDR--aR--E-WXGxyT^{171} where the first W^{154} is the Trp common to all Epds, and X is either Leu or Val. The residues close to the fourth common Cys are also well conserved showing a sequence of G^{209}I--p-VF-PPstC^{222}, where the Pro (P^{213}) has been substituted by Met in the echinoderm Epds. All the sequences from vertebrates that belong to the MERPs group have a Q^{77}RxRxL^{82} sequence four amino acids down from the common Asp (D^{73}). The X is a hydrophobic residue (Val, Ile or Leu). In non-vertebrate deuterostomes, only the sequence R^{78}xR^{80} is present, and other mostly hydrophobic residues are maintained in other positions.

The Basal group (Figure 5D) contains the only sequences where the common Trp (W^{136}) is substituted with a Tyr in Diplo_Tun and Oyster_Cg, or by a Phe in Oyster_Cv. As expected, sequences in this group also have several residues that are found within other groups. For example, all species have the first Arg found in the R^{56}xR^{58} sequence characteristic of the MERPs, but the second Arg is substituted by a Val in Aplysia and Biomphalaria. The Basal group also has the Tyr (Y^{86}) nine residues prior to the second Cys which is present in most other species with the exception of the vertebrate MERPS where a

Phe is in this position. In addition, the members of this Epd group share three amino acids in common positions: (i) the Gly (G^{117}) found 4 residues prior to the common Gly (G^{122}); (ii) the Asp (D^{184}) located seventeen residues prior to the final Cys; and (iii) the Phe (F^{195}) that is eleven residues after this Asp.

References used in additional files

1. **GenBank** [http://www.ncbi.nlm.nih.gov/]
2. **NCBI's Expressed Sequence Tags database (dbEST)** [http://www.ncbi.nlm.nih.gov/dbEST/]
3. **Ensembl genome browser** [http://www.ensembl.org/]
4. ***Tetraodon* Genome Browser** [http://www.genoscope.cns.fr/externe/tetranew/]
5. **NCBI's Sea Urchin Genome** [http://www.ncbi.nlm.nih.gov/projects/genome/guide/sea_urchin/]
6. **The Sea Urchin Gene Catalogue** [http://www.molgen.mpg.de/ag_seaurchin/]
7. **Medaka Expressed Sequence Tag Database** [http://mbase.bioweb.ne.jp/~dclust/medaka_top.html]
8. **Medaka Genome Browser at University of Tokyo** [http://medaka.utgenome.org/]
9. **NBRP Medakafish Genome Project by National Bio Resource Project (NBRP) group in National Institute of Genetics (Japan)** [http://shigen.lab.nig.ac.jp/medaka/genome/top.jsp]
10. **The Biology Workbench 3.2** [http://seqtool.sdsc.edu/CGI/BW.cgi]
11. **The CAP3 Sequence Assembly Machine** [http://bio.ifom-firc.it/ASSEMBLY/assemble.html]
12. Huang X: **An improved sequence assembly program.** *Genomics* 1996, **33:**21-31.
13. **NCBI's Open Reading Frame Finder (ORF finder)** [http://www.ncbi.nlm.nih.gov/gorf/gorf.html]
14. Schaffer AA, Aravind L, Madden TL, Shavirin S, Spouge JL, Wolf YI, Koonin EV, Altschul SF: **Improving the accuracy of PSI-BLAST protein database searches with composition-based statistics and other refinements.** *Nucleic Acids Res* 2001, **29:**2994-3005.
15. Altschul SF, Madden TL, Schäffer AA, Zhang J, Zhang Z, Miller W, Lipman DJ: **Gapped BLAST and PSI-BLAST: a new generation of protein database search programs.** *Nucleic Acids Res* 1997, **25:**3389-3402
16. Woods IG, Kelly PD, Chu F, Ngo-Hazelett P, Yan YL, Huang H, Postlethwait JH, Talbot WS: **A comparative map of the zebrafish genome.** *Genome Res* 2000, **10:**1903–1914.
17. Hubbard T, Andrews D, Caccamo M, Cameron G, Chen Y, Clamp M, Clarke L, Coates G, Cox T, Cunningham F, et al.: **Ensembl 2005.** *Nucleic Acids Res* 2005, **33:**D447–D453.
18. **InterPro database of protein families** [http://www.ebi.ac.uk/interpro/]
19. **NCBI's Conserved Domain Database (CDD)** [http://www.ncbi.nlm.nih.gov/Structure/cdd/wrpsb.cgi]
20. Marchler-Bauer A, Bryant SH: **CD-Search: protein domain annotations on the fly.** *Nucleic Acids Res* 2004, **32:**W327-331.

21. Ortí G, Meyer A: **Molecular evolution of ependymin and the phylogenetic resolution of early divergences among euteleost fishes.** *Mol Biol Evol* 1996, **13:**556-573.
22. Apostolopoulos J, Sparrow RL, McLeod JL, Collier FM, Darcy PK, Slater HR, Ngu C, Gregorio-King CC, Kirkland MA: **Identification and characterization of a novel family of mammalian ependymin-related proteins (MERPs) in hematopoietic, nonhematopoietic, and malignant tissues.** *DNA Cell Biol* 2001, **20:**625-35.
23. Suárez-Castillo EC, Medina-Ortíz WE, Roig-López JL, García-Arrarás JE: **Ependymin, a gene involved in regeneration and neuroplasticity in vertebrates, is overexpressed during regeneration in the echinoderm *Holothuria glaberrima*.** *Gene* 2004, **334:**133-143.
24. **SignalP 3.0 Server** [http://www.cbs.dtu.dk/services/SignalP/]
25. **NetNGlyc 1.0 Server** [http://www.cbs.dtu.dk/services/NetNGlyc]
26. **ExPASy ProtScale** [http://ca.expasy.org/cgi-bin/protscale.pl]
27. Kyte J, Doolittle RF: **A simple method for displaying the hydropathic character of a protein.** *J Mol Biol* 1982, **157:**105-132.
28. **Disulfind server** [http://disulfind.dsi.unifi.it/]
29. Vullo A, Frasconi P: **Disulfide connectivity prediction using recursive neural networks and evolutionary information.** *Bioinformatics* 2004, **20:**653-659.
30. **ProtParam tool** [http://ca.expasy.org/tools/protparam.html]
31. **The PredictProtein server** [http://www.embl-heidelberg.de/predictprotein]
32. **Sequence Search and Alignment by Hashing Algorithm (SSAHA tool in Ensembl)** [http://www.ensembl.org/Multi/blastview]
33. Boutet I, Tanguy A, Moraga D: **Response of the Pacific oyster *Crassostrea gigas* to hydrocarbon contamination under experimental conditions.** *Gene* 2004, **329:**147–157.
34. Poustka AJ, Groth D, Hennig S, Thamm S, Cameron A, Beck A, Reinhardt R, Herwig R, Panopoulou G, Lehrach H: **Generation, annotation, evolutionary analysis, and database integration of 20,000 unique sea urchin EST clusters.** *Genome Res* 2003, **13:**2736-2746
35. Satou Y, Yamada L, Mochizuki Y, Takatori N, Kawashima T, Sasaki A, Hamaguchi M, Awazu S, Yagi K, Sasakura Y, et al.: **A cDNA resource from the basal chordate *Ciona intestinalis*.** *Genesis* 2002, **33:**153-154.
36. Strausberg RL, Feingold EA, Grouse LH, Derge JG, Klausner RD, Collins FS, Wagner L, Shenmen CM, Schuler GD, Altschul SF, et al.: **Generation and initial analysis of more than 15,000 full-length human and mouse cDNA sequences.** *Proc Natl Acad Sci U S A* 2002, **99:**16899-16903.
37. Rise ML, von Schalburg KR, Brown GD, Mawer MA, Devlin RH, Kuipers N, Busby M, Beetz-Sargent M, Alberto R, Gibbs AR, et al.: **Development and application of a salmonid EST database and cDNA microarray: data mining and interspecific hybridization characteristics.** *Genome Res* 2004, **14:**478-90.
38. Tsoi SCM, Ewart KV, Penny S, Melville K, Liebscher RS, Brown LL, Douglas SE: **Identification of immune-relevant genes from Atlantic salmon using suppression subtractive hybridization.** *Mar Biotechnol* 2004, **6:**199-214.
39. Volz DC, Hinton DE, Law JM, Kullman SW: **Dynamic gene expression changes precede dioxin-induced liver pathogenesis in medaka fish.** *Toxicol Sci* 2006, **89:**524-534.

40. Renn SC, Aubin-Horth N, Hofmann HA: **Biologically meaningful expression profiling across species using heterologous hybridization to a cDNA microarray.** *BMC Genomics* 2004, **5:**42.
41. Clark MS, Edwards YJ, Peterson D, Clifton SW, Thompson AJ, Sasaki M, Suzuki Y, Kikuchi K, Watabe S, Kawakami K, et al.: **Fugu ESTs: new resources for transcription analysis and genome annotation.** *Genome Res* 2003, **13:**2747-2753.
42. Jaillon O, Aury JM, Brunet F, Petit JL, Stange-Thomann N, Mauceli E, Bouneau L, Fischer C, Ozouf-Costaz C, Bernot A, et al.: **Genome duplication in the teleost fish *Tetraodon nigroviridis* reveals the early vertebrate proto-karyotype.** *Nature* 2004, **431:**946-57.
43. Nielsen R, Bustamante C, Clark AG, Glanowski S, Sackton TB, Hubisz MJ, Fledel-Alon A, Tanenbaum DM, Civello D, White TJ, et al.: **A scan for positively selected genes in the genomes of humans and chimpanzees.** *PLoS Biol* 2005, **3:**E170.
44. Adams DS, Shashoua VE: **Cloning and sequencing the genes encoding goldfish and carp ependymin.** *Gene* 1994, **141:**237-241.
45. Konigstorfer A, Sterrer S, Hoffmann W: **Biosynthesis of ependymins from goldfish brain.** *J Biol Chem* 1989, **264:**13689-13692.
46. Adams DS, Kiyokawa M, Getman ME, Shashoua VE: **Genes encoding giant danio and golden shiner ependymin.** *Neurochem Res* 1996, **21:**377-384
47. Sterrer S, Konigstorfer A, Hoffmann W: **Biosynthesis and expression of ependymin homologous sequences in zebrafish brain.** *Neuroscience* 1990, **37:**277-284.
48. Muller-Schmid A, Rinder H, Lottspeich F, Gertzen EM, Hoffmann W: **Ependymins from the cerebrospinal fluid of salmonid fish: gene structure and molecular characterization.** *Gene* 1992, **118:**189-196.
49. Muller-Schmid A, Ganss B, Gorr T, Hoffmann W: **Molecular analysis of ependymins from the cerebrospinal fluid of the orders Clupeiformes and Salmoniformes: no indication for the existence of an euteleost infradivision.** *J Mol Evol* 1993, **36:**578-585.
50. Nimmrich I, Erdmann S, Melchers U, Chtarbova S, Finke U, Hentsch S, Hoffmann I, Oertel M, Hoffmann W, Muller O: **The novel ependymin related gene *UCC1* is highly expressed in colorectal tumor cells.** *Cancer Lett* 2001, **165:**71-79.
51. Aguileta G, Bielawski JP, Yang Z: **Gene conversion and functional divergence in the beta-globin gene family.** *J Mol Evol* 2004, **59:**177-189.
52. Nielsen R, Yang Z: **Likelihood models for detecting positively selected amino acid sites and applications to the HIV-1 envelope gene.** *Genetics* 1998, **148:**929–936.
53. Yang Z, Nielsen R, Goldman N, Pedersen AM: **Codon-substitution models for heterogeneous selection pressure at amino acid sites.** *Genetics* 2000, **155:**431–449.
54. Yang Z, Wong WSW, Nielsen R: **Bayes Empirical Bayes inference of amino acid sites under positive selection.** *Mol Biol Evol* 2005, **22:**1107-1118.
55. Yang Z, Nielsen R: **Codon-substitution models for detecting molecular adaptation at individual sites along specific lineages.** *Mol Biol Evol* 2002, **19:**908-917.
56. Zhang J, Nielsen R, Yang Z: **Evaluation of an improved branch-site likelihood method for detecting positive selection at the molecular level.** *Mol Biol Evol* 2005, **22:**2472-2479.
