# Supplementary material for: Molecular evolution of the ependymin protein family: a necessary update
Source: BMC Evol Biol. 2007 Feb 15;7:23. doi: 10.1186/1471-2148-7-23 (PMC1805737; doi:10.1186/1471-2148-7-23)
Supplement: Additional file 5 — Statistical Analyses of Selective Pressure. Parameter estimates of ω ratios, log-likelihood scores and LRT statistics for the ependymin data under different branch-, site-, and branch-site models of codon evolution. [file 1471-2148-7-23-S5.pdf]

Table S4. Parameter estimates of ω ratios, log-likelihood scores and LRT statistics for the ependymin data under different branch-, site-, and branch-site models of codon evolution. (References provided in the Additional file 2: Additional_Text.pdf)

Model	Estimates of Parameters	ℓ [a]	LRT [b]	df [c]	*p* Value	PSS [d]
M0: $\omega_o = \omega_{FishBrain} = \omega_{FishTj} = \omega_{MERPs}$	$\omega_o = 0.155$	-27658.44				None
**Postduplication and Postspeciation (PD-PS) Models** [51]						
Mps₁: ω_b; $\omega_{MERPs(PD)} = \omega_{FishBrain(PD)} = \omega_{FishTj(PD)}$; $\omega_{MERPs(PS)} = \omega_{FishBrain(PS)} = \omega_{FishTj(PS)}$	$\omega_{(PD)} = 1.139$, $\omega_{(PS)} = 0.161$, $\omega_b = 0.056$	-27650.30	M0 vs. Mps_1	2	0.0003	
Mps₂: $\omega_b = \omega_{MERPs}$; $\omega_{FishBrain(PD)} = \omega_{FishTj(PD)}$; $\omega_{FishBrain(PS)} = \omega_{FishTj(PS)}$	$\omega_{(PD)} = 1.983$, $\omega_{(PS)} = 0.180$, $\omega_b = 0.114$	-27644.73	M0 vs. Mps_2	2	< 0.0001	
**Paralog Models** [51]						
Mp₁: ω_b; $\omega_{FishBrain}$; ω_{FishTj}; ω_{MERPs}	$\omega_{FishBrain} = 0.175$; $\omega_{FishTj} = 0.198$; $\omega_{MERPs} = 0.123$; $\omega_b = 0.057$	-27641.01	M0 vs. Mp_1	3	< 0.0001	
Mp₂: $\omega_b = \omega_{MERPs}$; $\omega_{FishBrain}$; ω_{FishTj}	$\omega_{FishBrain} = 0.175$; $\omega_{FishTj} = 0.198$; $\omega_{MERPs} = \omega_b = 0.114$	-27644.89	M0 vs. Mp_2	2	< 0.0001	
Mp₃: ω_b; ω_{MERPs}; $\omega_{Non\text{-}MERPs}$	$\omega_{MERPs} = 0.1221$; $\omega_{Non\text{-}MERPs} = 0.1793$; $\omega_b = 0.068$	-27644.35	M0 vs. Mp_3	2	< 0.0001	
**Site - Models (Separate groups)** [52-54]						
Model 0: One-ratio						
FishBrain (32 Taxa)	$\omega_o = 0.182$	-9390.26				
FishTj (12 Taxa)	$\omega_o = 0.195$	-6681.65				
MERPs (17 Taxa)	$\omega_o = 0.115$	-6147.06				
Basal (8 Taxa)	$\omega_o = 0.051$	-5050.18				

Model 3: Discrete (K = 3)						
FishBrain (32 Taxa)	$\omega_o = 0.004$, $p_o = 0.099$, $\omega_1 = 0.114$, $p_1 = 0.498$, $\omega_2 = 0.414$, ($p_2 = 0.403$)	-9215.15	M0 vs. M3 (K =3)	4	< 0.0001	None
FishTj (12 Taxa)	$\omega_o = 0.014$, $p_o = 0.184$, $\omega_1 = 0.153$, $p_1 = 0.415$, $\omega_2 = 0.470$, ($p_2 = 0.402$)	-6530.63	M0 vs. M3 (K =3)	4	< 0.0001	None
MERPs (17 Taxa)	$\omega_o = 0.009$, $p_o = 0.331$, $\omega_1 = 0.090$, $p_1 = 0.418$, $\omega_2 = 0.482$, ($p_2 = 0.251$)	-5929.25	M0 vs. M3 (K =3)	4	< 0.0001	None
Basal (8 Taxa)	$\omega_o = 0.003$, $p_o = 0.120$, $\omega_1 = 0.059$, $p_1 = 0.757$, $\omega_2 = 0.382$, ($p_2 = 0.122$)	-4972.79	M0 vs. M3 (K =3)	4	< 0.0001	None
**Site - Models (70 sequences dataset)** [52-54]						
Model 0: one-ratio	$\omega_o = 0.155$	-27658.44				None
Model 1a: Nearly Neutral (K = 2)	($\omega_o = 0.155$), $p_o = 0.818$, ($\omega_1 = 1$), ($p_1 = 0.182$)	-27729.28				Not Allowed
Model 3: Discrete (K = 2)	$\omega_o = 0.0834$, $p_o = 0.369$, $\omega_1 = 0.268$, ($p_1 = 0.631$)	-27471.28	M0 vs. M3 (K = 2)	2	< 0.0001	None
Model 3: Discrete (K = 3)	$\omega_o = 0.035$, $p_o = 0.121$, $\omega_1 = 0.137$, $p_1 = 0.430$, $\omega_2 = 0.320$, ($p_2 = 0.448$)	-27402.19	M0 vs. M3 (K = 3)	4	< 0.0001	None
**Branch-Site Models (Testing PD branches for positive selection)** [54-56]			**PSS [d] for each lineage Prob (ω>1) using BEB inference**			
Model A:						
FishBrain	$p_o = 0.396$, $p_1 = 0.087$, ($p_2 + p_3 = 0.517$), $\omega_2 = \infty$	-27724.32	**51W** (p = 0.975), **126Q** (p = 0.951), **186 _** (p = 0.952)			
FishTj	$p_o = 0.790$, $p_1 = 0.174$, ($p_2 + p_3 = 0.036$), $\omega_2 = \infty$	-27726.70	**144S** (p = 0.643)			
MERPs	$p_o = 0.587$, $p_1 = 0.127$, ($p_2 + p_3 = 0.285$), $\omega_2 = \infty$	-27717.96	**86R** (p = 0.964), **128W** (p = 0.993), **196T** (p = 0.972), **224S** (p = 0.973), **231T** (p = 0.952)			

Model B:						
FishBrain	$p_o = 0.257$, $p_1 = 0.436$, ($p_2 + p_3 = 0.307$), $\omega_o = 0.083$, $\omega_1 = 0.270$, $\omega_2 = \mathbf{1.983}$	-27466.48	MB vs. M3 (K = 2)	2	0.0083	Model B fits the data better than M3 (K=2)
FishTj	$p_o = 0.280$, $p_1 = 0.477$, ($p_2 + p_3 = 0.243$), $\omega_o = 0.084$, $\omega_1 = 0.268$, $\omega_2 = \mathbf{2.420}$	-27471.28	MB vs. M3 (K = 2)	2	1.0	N.S.
MERPs	$p_o = 0.315$, $p_1 = 0.530$, ($p_2 + p_3 = 0.155$), $\omega_o = 0.084$, $\omega_1 = 0.270$, $\omega_2 = \mathbf{18.170}$	-27469.29	MB vs. M3 (K = 2)	2	0.1372	N.S.
Branch-Site Tests of Positive Selection			**Branch-site TEST 1[e]**		**Branch-site TEST 2[e]**	
FishBrain (MA with $\omega_2 = 1$ Fixed)	$p_o = 0.422$, $p_1 = 0.094$, ($p_2 + p_3 = 0.484$), $\omega_o = 0.155$, $\omega_1 = 1$, $\omega_2 = 1$	-27725.91	MA vs. M1a df = 2	$p = 0.0071$	MA vs. MA ($\omega_2 = 1$) df = 1	$\boldsymbol{p} = \mathbf{0.0747}$
FishTj (MA with $\omega_2 = 1$ Fixed)	$p_o = 0.741$, $p_1 = 0.165$, ($p_2 + p_3 = 0.094$), $\omega_o = 0.155$, $\omega_1 = 1$, $\omega_2 = 1$	-27729.28	MA vs. M1a df = 2	$p = 0.0763$	MA vs. MA ($\omega_2 = 1$) df = 1	$\boldsymbol{p} = \mathbf{0.0233}$
MERPs (MA with $\omega_2 = 1$ Fixed)	$p_o = 0.570$, $p_1 = 0.124$, ($p_2 + p_3 = 0.306$), $\omega_o = 0.154$, $\omega_1 = 1$, $\omega_2 = 1$	-27725.42	MA vs. M1a df = 2	$p < 0.0001$	MA vs. MA ($\omega_2 = 1$) df = 1	$\boldsymbol{p} = \mathbf{0.0001}$

[a] ℓ, Log-likelihood scores.

[b] LRT, likelihood ratio test.

[c] df, Degrees of freedom.

[d] PSS, Positively Selected Sites. Sites potentially under positive selection are identified using the human ependymin sequence [Epdr_Homo] as the reference.

[e] TEST 1 and TEST 2 as defined in the Methods section of the main text [54-56]
